# MEMOIR-VLM—a multimodal vision-language model for Alzheimer's disease classification and question answering

**DOI:** 10.3389/fncom.2026.1902258

**Published:** 2026-10-01

**Authors:** Sohail Haresh Gidwani, Tamoghna Chattopadhyay, Sophia I. Thomopoulos, Paul M. Thompson

**Affiliations:** Imaging Genetics Center, Mark and Mary Stevens Neuroimaging and Informatics Institute, Keck School of Medicine, University of Southern California, Marina del Rey, CA, United States

**Keywords:** Alzheimer's disease, deep learning, missing data, vision-language model, retrieval-augmented generation, visual question answering, diffusion tensor imaging, large language model

## Abstract

**Introduction:**

Alzheimer's disease (AD) affects an estimated 55 million people worldwide and is projected to nearly double by 2050, creating a need for scalable, non-invasive AI systems that can classify disease stage, support multimodal clinical reasoning, and interact in natural language. Most deep learning work on AD focuses on single-modality classification without natural-language interaction and typically assumes complete input data.

**Methods:**

We present MEMOIR-VLM (Multimodal Encoder with Missing-modality and Open-ended Inference for Retrieval), a modular two-stage vision-language framework trained and evaluated on 2,363 ADNI subjects. The first stage is a missing-modality-aware encoder that fuses T1-weighted MRI and DTI fractional anisotropy maps with structured clinical scores via cross-attention fusion with stochastic modality dropout, allowing a single set of weights to operate on any of the seven non-empty modality subsets without imputation. After CLIP/InfoNCE contrastive pretraining, the encoder is fine-tuned for multi-task prediction of 3-way diagnosis, binary diagnosis, CDR-SB, age, and sex. The second stage extends the frozen encoder into a retrieval-augmented generation pipeline for case-based reasoning and open-ended visual question answering.

**Results:**

On held-out ADNI test data, with CDR-SB excluded from the inputs to remove a cognitive-score leak, the encoder achieved 91.3% balanced accuracy for CN vs. dementia and 68.2% balanced accuracy for 3-class diagnosis (macro-F1 = 0.681); age was predicted with a 5.96-year MAE. When the diagnosis field was withheld from retrieved captions, no evaluated LLM exceeded a retrieval-only k-NN majority vote (0.673), which itself closely matched the encoder's 3-class accuracy (0.682). The RAG-VQA layer showed strong text fidelity (BERTScore = 0.894; SBERT cosine similarity = 0.811). Zero-shot external validation on OASIS-3 (1,048 subjects, no diffusion data) retained 78.7% balanced accuracy for CN vs. impaired (AUC = 0.889) using identical ADNI-trained weights with the DTI branch masked.

**Discussion:**

MEMOIR-VLM combines missing-modality-aware multimodal encoding, multi-task clinical prediction, and retrieval-grounded natural-language reasoning within a single modular framework. The results support using the encoder, optionally with k-NN voting, as the diagnostic component and the RAG-VQA layer as an interpretable natural-language interface that surfaces comparable cases and readable clinical summaries. The external validation results further demonstrate the practical value of the missing-modality mechanism when an imaging modality is unavailable.

## 1 Introduction

Alzheimer's disease (AD) is the most common cause of dementia and one of the most pressing public health crises of the twenty-first century. Dementia is currently the seventh leading cause of death and one of the major causes of disability and dependency among older people globally (World Health Organization, 2020). An estimated 57.4 million individuals were living with dementia worldwide in 2019, a figure projected to rise to 152.8 million by 2050 (GBD 2019 Dementia Forecasting Collaborators, 2022). Much of this increase will be concentrated in low- and middle-income countries (LMICs) as already 60% of people with dementia live in LMICs, and by 2050 this proportion is expected to reach 71%, with the fastest growth occurring in China, India, and their South Asian and Western Pacific neighbors (Alzheimer's Disease International, n.d.). The human and economic costs are equally alarming. In 2019, dementia cost economies globally US$1.3 trillion, with approximately 50% of these costs attributable to informal carers who provide on average 5 h of care and supervision per day (World Health Organization, 2020). The global cost is projected to increase to US$1.7 trillion by 2030, or US$2.8 trillion if corrected for increases in care costs (World Health Organization, 2020). The burden is also deeply inequitable as women experience higher disability-adjusted life years and mortality due to dementia, but also provide 70% of care hours for people living with dementia (World Health Organization, 2020). Despite the scale of this crisis, access to timely diagnosis and care remains severely limited. In high-income countries, only 20%−50% of dementia cases are recognized and documented in primary care, while in LMICs, one study in India suggested that 90% of cases remain undiagnosed implying that approximately three-quarters of all people with dementia worldwide have not received a formal diagnosis (Seeher et al., 2023). Given this situation, scalable systems are needed for early detection and monitoring of people with mild cognitive impairment and dementia. Artificial intelligence systems can assist with reproducible, and interpretable radiological assessment, by helping to analyze and make inferences from commonly collected clinical and radiologic data, with considerable value in clinical practice.

Alzheimer's disease is fundamentally a multimodal condition. Its clinical presentation includes macrostructural brain atrophy visible on T1-weighted (T1w) brain MRI, and white matter microstructural deterioration quantifiable with diffusion tensor imaging (DTI) *via* measures such as the fractional anisotropy (FA) of diffusion, which tends to be reduced as the brain deteriorates. AD progression is defined in research contexts (Jack et al., 2016, 2018) in terms of amyloid and tau pathology measurable through PET or CSF biofluid assays, and cognitive decline is assessed with standardized neuropsychological batteries such as the MMSE (Folstein et al., 1975), ADAS-13 (Kueper et al., 2018), MoCA (Hobson, 2015), and CDR-SB (O'Bryant et al., 2008). Genetic risk for late-onset AD can be assessed based on a person's APOE genotype (Mayeux et al., 1998) although genetic risk is not routinely assessed in the clinical setting, and is more commonly measured in research settings and some clinical trials.

As can be seen from the wide range of data types routinely collected in the clinical evaluation of a person with probable AD, no single data stream fully characterizes the disease—structural MRI captures neurodegeneration but not molecular pathology (except indirectly); cognitive scores reflect current function but not underlying biology; DTI maps white matter integrity but is less universally available. The clinical gold standard for Alzheimer's assessment integrates as many of these dimensions as are available.

The Alzheimer's Disease Neuroimaging Initiative (ADNI; Jack et al., 2008) was established to validate biomarkers of AD, and prioritize those that are currently available, by assessing one of the largest longitudinal cohorts of cognitively healthy, MCI, and dementia participants with MRI, PET, CSF, clinical, and multi-omics data. This rich multimodal dataset catalyzed a generation of machine learning studies, but most examine only a single clinical endpoint, and lack any means to deal with question answering in natural language, which can be more valuable in real-world clinical situations.

Open-source platforms such as Clinica have supported standardized and reproducible processing and analysis of clinical neuroimaging datasets (Routier et al., 2019). For analyzing brain images in particular, AI methods have greatly advanced over the past decade. Multimodal deep learning approaches have further demonstrated the value of integrating complementary imaging and clinical information for Alzheimer's disease diagnosis (Golovanevsky et al., 2022; Rahman et al., 2025). Convolutional neural networks (CNNs; Chattopadhyay et al., 2023b, 2024a,b), recurrent neural networks (Mehdipour Ghazi et al., 2019; Petrosian et al., 2001; Wang et al., 2018), and transformers (Chattopadhyay et al., 2024a; Dhinagar et al., 2023, 2025b) now come close to clinical expert levels in a range of tasks, including AD classification and predicting MCI conversion (Chattopadhyay et al., 2025d, 2026; Marinescu et al., 2019) from brain MRI. Deep learning models have also been investigated for predicting Alzheimer's disease biomarkers, including amyloid positivity, directly from structural T1-weighted MRI Chattopadhyay et al., 2024c. More recently, large language models (LLMs; Chang et al., 2024; Zhao et al., 2023; Thirunavukarasu et al., 2023) and vision-language models (VLMs; Jeong et al., 2024; Hartsock and Rasool, 2024; Zhang J. et al., 2024) have begun to combine learned visual representations with other kinds of clinical data. Such systems can respond to questions about biomedical input data in natural language (visual question answering, or VQA), and they can also be incorporated into larger agentic systems to perform specific sets of tasks such as binary or multi-class classification, differential diagnosis and staging of the illness.

Systems such as GPT-4V (Wu et al., 2023; Yang et al., 2023), LLaVA (Liu et al., 2023), PaliGemma2 (Steiner et al., 2024), BioMedGPT (Zhang K. et al., 2024), MedBLIP (Chen and Hong, 2024), NeuroBERT (Wood et al., 2024) and Med-PaLM (Singhal et al., 2025; Tu et al., 2024) show that LLMs can reason over clinical images when coupled with appropriate visual encoders. To draw from a wider range of data to support inferences, LLMs and VLMs can use retrieval-augmented generation (RAG; Gao et al., 2023; Lewis et al., 2020), which informs LLM outputs using evidence retrieved from a reference database, providing traceable evidence chains that are important for clinical trust.

With some recent exceptions (Dhinagar et al., 2026a,b; Jasodanand et al., 2025a,b; Jia et al., 2026; Lteif et al., 2026), the application of RAG and VLMs to multimodal neuroimaging is only starting to be attempted. Most clinical AI systems that perform tasks relevant to AD predominantly function as discriminative classifiers that output a categorical label or probability score, without providing natural-language explanations. There is great interest in AI methods that provide open-ended answering capabilities so that clinicians can integrate AI predictions into their reasoning and communicate findings to patients and families. A growing body of work has begun to apply vision-language models (VLMs), visual question answering (VQA) systems, and large language models (LLMs) to medical imaging and, more recently, to neuroimaging. Chen et al. (Chen and Hong, 2024) introduced MedBLIP, a vision-language foundation model that integrates 3D volumetric MRI with electronic health records *via* a MedQFormer cross-modal alignment module and a BioMedLM language backbone, showing it is feasible to combine 3D brain images with free-text clinical descriptions. While MedBLIP supports report generation and a form of VQA, its outputs are purely generative text and it does not perform structured clinical prediction tasks such as multi-class diagnosis, amyloid positivity classification, or cognitive score regression. Wu et al. (2025) proposed RadFM, the first general radiology foundation model able to process both 2D and 3D scans using a 3D Vision Transformer (ViT). In this approach, a perceiver module compresses 3D image features into a compact token sequence, achieving an F1 value of 78.1 on the VQA-RAD (Lau et al., 2018) benchmark. (Li et al., 2023) extended the LLaVA architecture to the medical domain through biomedical instruction tuning on PubMed Central image-text pairs, enabling open-ended medical visual question answering in a conversational format. Wang et al. (2026) evaluated ten LLMs, including DeepSeek-R1, GPT-4, and clinical fine-tuned variants, for the task of generating diagnostic impressions from brain MRI. Their top-performing system (DeepSeek-R1) achieved 97.6% patient-level accuracy using a differential-diagnosis prompting strategy and reduced radiologist reading time, showing that LLMs can aid in structured neurological reporting. Lin et al. (2025) proposed ADLIP, a vision-language model for AD diagnosis built on CLIP-style contrastive pretraining and multi-teacher knowledge distillation to resolve ambiguous diagnostic boundaries between CN, MCI, and AD. ADLIP achieved strong 3-class classification and zero-shot generalization to an external ancestrally diverse cohort, but it does not support natural-language output, retrieval of similar cases, or open-ended clinical querying. Can et al. (2025b) introduced VisTA, a contrastive vision-text alignment framework for Alzheimer's diagnosis that retrieves reference cases from an expert-annotated database to generate evidence-driven predictions and explanations of abnormalities. Can et al. (2025a) presented REMEMBER (Retrieval-based Explainable Multimodal Evidence-guided Modeling for Brain Evaluation and Reasoning), which trains a contrastively aligned vision-text model on expert-annotated reference data, retrieves similar MRI cases at inference time, and integrates their contextual information through an attention-based inference head to produce zero- and few-shot diagnoses with interpretable reports. Gupta et al. (2025) proposed BRAINS (Biomedical Retrieval-Augmented Intelligence for Neurodegeneration Screening), a dual-module system combining a cognitive diagnostic module (an LLM fine-tuned on MMSE and CDR scores) with a case retrieval module that encodes patient profiles into latent representations and retrieves similar cases from a curated knowledge base. Dhinagar et al. (2025a) proposed a VLM that aligns 3D T1w scans with clinical text captions using a two-stage pipeline: unimodal pretraining (MLM on clinical text, supervised pretraining of a 3D DenseNet-121 image encoder) followed by cross-modal contrastive fine-tuning. The resulting system supports MRI retrieval, automated captioning, classification, and visual question answering.

Across this rapidly developing body of VLM, VQA, and LLM work applied to neuroimaging, there are several common limitations and challenges. Many systems cannot deal with missing modalities, especially when using a multimodal encoder with a generative VQA pipeline: existing approaches either provide robust image encoding or retrieval-augmented LLM generation, but not both in a unified framework that handles any available subset of imaging and clinical data. Also no published VLM or VQA system in AD neuroimaging performs multi-task clinical prediction, inferring cognitive severity and diagnosis. MEMOIR-VLM is designed to simultaneously address all such gaps.

The individual components of MEMOIR-VLM (cross-attention fusion, CLIP-style contrastive alignment, FAISS retrieval with cross-encoder reranking, and instruction-tuned language generation) are each established in the literature, including in several of the works cited above, and we do not claim these components as an original contribution in our current work. Our contribution is one of capability coverage rather than methodological invention: to our knowledge, existing systems provide either robust multimodal encoding or retrieval-augmented language generation, but not both within a single framework whose weights operate on any available subset of imaging and clinical inputs. MEMOIR-VLM combines a per-sample modality mask admits any available combination of the three input modalities (T1w, DTI-FA, and clinical), that is, all seven non-empty subsets, from any single modality to the full set, without imputation and without a separate model per subset, with multi-task clinical prediction and a retrieval-grounded natural-language interface, all supported by one shared 512-dimensional representation. Table 1 situates MEMOIR-VLM relative to these systems along capability axes.

**Table 1:** Positioning of MEMOIR-VLM relative to closely related multimodal and vision–language systems for Alzheimer's disease.

System	Imaging input	Non-imaging input	Missing modalities handled without imputation	Multi-task clinical prediction	Natural-language output	Case retrieval
MedBLIP (Chen and Hong, 2024)	3D MRI	EHR text	No	No (generative only)	Yes	No
ADLIP (Lin et al., 2025)	3D T1w MRI	Structured clinical	No	Partial (DX + MMSE)	No	No
VisTA (Can et al., 2025b)	MRI	Expert annotations	No	No (DX + explanation)	Yes	Yes (expert -annotated DB)
REMEMBER (Can et al., 2025b)	MRI	Expert annotations	No	No (DX + report)	Yes	Yes
BRAINS (Gupta et al., 2025)	None (profile-based)	MMSE, CDR	No	No	Yes	Yes
MEMOIR-VLM (our work)	T1w MRI + DTI-FA	Clinical + APOE	Yes (per-sample mask; all subsets, one weight set)	Yes (DX2, DX3, sex, age, CDR-SB)	Yes (captioning + VQA)	Yes (FAISS + cross-encoder)

Capability columns are verifiable from the source publications. DX2 is CN vs. Dem Classification and DX3 is CN vs. MCI vs. Dem Classification.

A specific and underappreciated challenge in clinical neuroimaging AI is incomplete real-world data. Although cohorts such as Alzheimer's Disease Neuroimaging Initiative collect multiple imaging modalities, many subjects in clinical and research settings lack complete data. DTI is often missing as it requires longer scan times, is sensitive to motion and susceptibility artifacts, and is not always available in standard clinical imaging protocols. Cognitive battery completion is similarly variable; as is amyloid PET assessment (Camus et al., 2012; Johnson et al., 2013), a key biomarker in AD research but less widely available at typical dementia care clinics. Some multimodal deep learning methods address incomplete imaging data by imputing synthetic data with generative models such as GANs or diffusion models (Chattopadhyay et al., 2025a,b,c; Zhang et al., 2025) or by restricting training and evaluation to the subset of subjects with complete imaging data, sacrificing sample size and generalizability. Neither approach is ideal for clinical deployment as generative synthesis of missing imaging modalities introduces hallucination risk and requires a separate model per modality, while complete-case analysis fails to reflect the distribution of patients a deployed system will encounter. A robust clinical AI system needs to produce predictions from whichever combination of modalities is available for a given individual, without deteriorating severely when a modality is absent.

In the current paper, we present MEMOIR-VLM (Multimodal Encoder with Missing-modality and Open-ended Inference for Retrieval), a two-stage framework designed to address the limitations above. In the first stage, a missing-modality-aware deep learning encoder jointly learns from T1w MRI, DTI FA maps, and structured clinical scores to simultaneously predict five clinically meaningful targets - 3-way diagnosis (CN/MCI/Dementia), binary diagnosis (CN vs. Dementia), biological sex, age, and CDR-SB severity. Sex and age are included as benchmarking tasks and to help train the encoder, as ground truth is known. The encoder handles any subset of available input modalities through a cross-attention fusion mechanism with stochastic modality dropout, requiring no imputation and no separate model for different subsets of modalities. In the second stage, the frozen encoder is extended *via* retrieval-augmented generation to enable natural-language case-based reasoning, automated caption generation, and open-ended visual question answering over unseen brain scans.

## 2 Imaging data and preprocessing

### 2.1 Dataset

All neuroimaging and clinical data used in this study were obtained from the Alzheimer's Disease Neuroimaging Initiative [ADNI (Jack et al., 2008)], a multisite longitudinal study launched in 2004 with the goal of developing validated biomarkers for Alzheimer's disease clinical trials. ADNI has enrolled participants across three diagnostic categories; cognitively normal (CN), mild cognitive impairment (MCI), and dementia. The rich data in ADNI from brain MRI, PET, CSF, genetic, and neuropsychological testing, make it one of the most comprehensive publicly available neuroimaging resources for AD research.

The full dataset comprises 2,363 unique subjects drawn from ADNI, filtered to individuals with valid diagnostic labels and 3D T1-weighted brain MRI. Because ADNI is longitudinal and multi-phase, the cohort was deduplicated to one scan per subject prior to any partitioning, retaining the most recent available scan date; each subject therefore contributes exactly one row, and no longitudinal replicates of the same individual exist anywhere in the dataset. The 80/20 split was then performed at the subject level, stratified by diagnostic class, yielding a training set of 1,889 subjects (669 CN, 650 MCI, 570 Dementia) and a held-out test set of 474 subjects (168 CN, 163 MCI, 143 Dementia). We verified that the intersection of training and test subject identifiers is empty: no subject appears in both partitions, and the scan-level leakage that arises when repeated visits of the same individual are distributed across partitions is structurally precluded. The same discipline extends downstream: the FAISS reference index is built exclusively from the 1,889 training subjects, so a test subject cannot retrieve itself, and the text encoder's contrastive alignment is fitted only on training-subject pairs. Figure 1 shows the diagnosis distribution across train/test splits. The demographics are described in Table 2.

**Figure 1:**
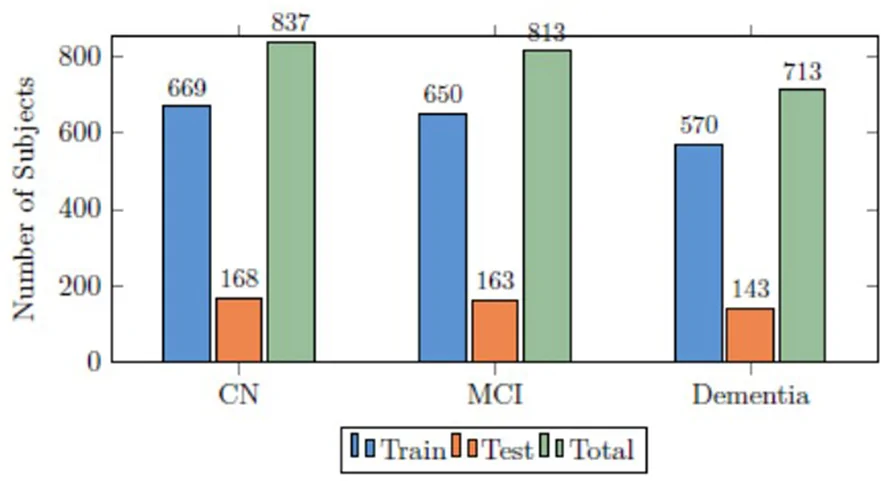
Diagnosis distribution across train/test splits. The dataset is well-balanced across all three classes.

**Table 2:** Demographics details of the dataset.

Demographic characteristic	Train (*n* = 1,889)	Test (*n* = 474)
Age (mean ± SD)	75.9 ± 7.7	75.5 ± 7.8
Age range	55.1–97.4	55.2–95.4
Sex (M/F)	978/911	260/214
Sex ratio (%M)	51.8%	54.9%

For external validation we additionally use OASIS-3 (Open Access Series of Imaging Studies; LaMontagne et al., 2019), comprising 1,048 subjects (one session per subject; 751 CN, 297 impaired). OASIS-3 provides T1-weighted MRI and structured clinical scores but no processed diffusion maps, so DTI-FA is absent for all external subjects. No OASIS-3 data were used at any stage of training, model selection, or index construction; the cohort is held out entirely and used only for zero-shot evaluation. Both ADNI and OASIS-3 are governed by their respective Data Use Agreements; no subject identifiers, images, or tabular records are redistributed, and all reported values are aggregate metrics.

### 2.2 Modality availability

Diffusion MRI (dMRI) data were available for a subset of subjects. DTI scalar maps were derived from ADNI GO/2 and ADNI3 data. In total, 930 participants (39.4% of the full cohort) had usable DTI data: 751 in the training set (39.8%) and 179 in the test set (37.8%). Table 3 describes the modality availability across train/test splits.

**Table 3:** Modality availability across splits.

Modality	Train		Test	
	Count	%	Count	%
T1w MRI	1,889	100.0%	474	100.0%
DTI-FA maps	751	39.8%	179	37.8%
Clinical features	~1,889	~100%	~474	~100%

In addition to imaging, structured clinical and biomarker features were extracted for each subject. Features used as model inputs were limited to variables that do not constitute prediction targets, to prevent data leakage. The clinical encoder receives four continuous variables: ADAS-11, ADAS-13, MMSE, and MoCA, together with APOE genotype as a categorical input. Availability varied across features: MMSE was present for 91.2% of training subjects, ADAS-11 for 90.3%, ADAS-13 for 89.3%, and APOE genotype for 91.5%, while MoCA was available for 63.9%. Missing scalar feature values were imputed with population-level means during training and inference; this applies only to individual clinical scores (e.g., a missing MoCA value) and is distinct from imaging modality imputation, which MEMOIR-VLM is explicitly designed to avoid.

### 2.3 T1-weighted MRI preprocessing

All T1-weighted MRI volumes underwent a standardized preprocessing pipeline (Chattopadhyay et al., 2023a; Lam et al., 2020) prior to model training. Processing steps were applied uniformly across all ADNI datasets and acquisition sites to ensure consistent spatial and intensity characteristics:

*Bias field correction:* Intensity non-uniformity arising from radiofrequency field inhomogeneities was corrected using N4 bias field correction, which fits a smooth multiplicative bias field to each volume and removes low-frequency intensity gradients that would otherwise confound downstream feature extraction.

*Skull stripping:* Brain tissue was isolated by removing non-brain structures (such as skull, scalp, dura, and background) using automated “skull stripping”, producing a brain-only mask that eliminates irrelevant intensity variation from non-brain tissue.

*Spatial registration:* Each skull-stripped volume was nonlinearly registered to a common in-house anatomical template using a 9-degree-of-freedom (9DOF) transformation, accounting for translation, rotation, and scaling while preserving local anatomical morphology. Registration to a shared template ensures that anatomically homologous regions occupy the same voxel coordinates across all subjects, enabling the 3D convolutional encoder to learn spatially consistent features.

*Resampling:* Following registration, all volumes were resampled to isotropic 2 mm resolution, yielding uniform image dimensions of 91 × 109 × 91 voxels across the entire dataset.

*Intensity normalization:* Voxel intensities were normalized to the [0, 1] range using min–max scaling, computed independently per volume, to reduce inter-subject and inter-site intensity variability and to facilitate stable gradient-based optimization during deep learning model training.

### 2.4 Diffusion tensor imaging preprocessing

Diffusion MRI preprocessing followed established ADNI harmonization protocols, with cohort-specific adaptations. For ADNI GO/2 acquisitions, the standard ADNI dMRI preprocessing (Nir et al., 2013; Zavaliangos-Petropulu et al., 2019) pipeline was applied. For ADNI3 acquisitions, an updated pipeline (Thomopoulos et al., 2021) was used to account for scanner hardware and acquisition protocol variability introduced by the transition to newer MRI systems across sites.

Diffusion tensor models were fit to the preprocessed dMRI data using FSL's DTIFIT (Jenkinson et al., 2012; Woolrich et al., 2009), from which four scalar maps were derived per subject: fractional anisotropy (FA), axial, mean and radial diffusivity (AxD/MD/RD). FA maps quantify the directional coherence of water diffusion—a sensitive marker of white matter microstructural integrity; FA was used as the primary DTI input for the MEMOIR-VLM encoder. All DTI scalar maps were resampled to 2 mm isotropic resolution, yielding spatial dimensions of 91 × 109 × 91 voxels, and were spatially aligned to the ENIGMA TBSS DTI template (Jahanshad et al., 2013) using tract-based spatial statistics (TBSS). This ensures that the DTI data is aligned with the T1 registration template and enables direct integration of T1 and DTI feature maps within the cross-attention fusion module.

## 3 Models and experiments

### 3.1 System overview

MEMOIR-VLM is a two-stage system for Alzheimer's Disease (AD) characterization from multimodal neuroimaging and clinical data, with capacity for predictive modeling and interpretable reasoning. The framework integrates a missing-modality-aware multimodal encoder with a retrieval-augmented generation pipeline, to make structured predictions and more open-ended text responses. In the first stage, a multimodal encoder processes three heterogeneous input modalities; T1w, DTI-FA, and structured clinical features; and jointly predicts multiple clinically relevant targets. This stage learns a robust shared representation under incomplete modality conditions. In the second stage, a retrieval-augmented visual question answering (VQA) pipeline extends the frozen encoder with language capabilities, enabling natural-language interaction with brain scans. Together, these two stages instantiate the “Locked Image Tuning” paradigm, in which imaging and clinical encoders are trained end-to-end and subsequently frozen, allowing downstream reasoning modules to operate entirely in the learned embedding space without modifying the underlying visual representations. The resulting system supports three clinically meaningful applications: (i) retrieval of similar historical cases from a reference cohort, (ii) automated generation of natural-language clinical descriptions, and (iii) open-ended question answering grounded in retrieved evidence. The system components are summarized in Table 4.

**Table 4:** System components and specifications.

Component	Type	Specifications
T1w encoder	ResNet-18 3D	Input: (1, 91, 109, 91); Output: 512-d embedding
DTI-FA encoder	ResNet-18 3D	Same architecture as T1w encoder; separate weights
Clinical encoder	MLP	6 continuous features + APOE genotype embedding → 512-d
Attention fusion	Cross-Attention	8-head attention, learnable pool query, per-modality masking gates
Task heads	MLP ( × 6)	512 → 256 → 128 → K with dropout; one head per prediction target
Text encoder	all-MiniLM-L6-v2	22.7M params; MLM pre-trained on 26,889 clinical sentences; 384-d output
Text projection	MLP	384 → 512 with LayerNorm, GELU, Dropout; 462K trainable parameters
FAISS index	IndexFlatIP	Exact inner-product (cosine) search on L2-normalized 512-d vectors; 1,889 training subjects
Cross-encoder	ms-marco-MiniLM-L-6-v2	Pairwise relevance scoring; reranks top-20 → top-5 candidates
LLM	Mistral-7B-Instruct-v0.3	4-bit NF4 quantization (BitsAndBytes); ~4 GB VRAM; greedy decoding, max 200 tokens

### 3.2 Multimodal encoder architecture

The multimodal encoder forms the core of MEMOIR-VLM, transforming heterogeneous inputs into a unified latent representation that is robust to missing data and valuable for multiple prediction tasks. The architecture consists of modality-specific encoders, an attention-based fusion mechanism, and a set of multi-task prediction heads.

#### 3.2.1 Modality specific encoders

Each modality is processed using a dedicated encoder tailored to its structure and statistical properties (Figure 2). T1w MRI and DTI-FA volumes are encoded using independent 3D ResNet-18 (Ayyachamy et al., 2019) networks, each receiving a single-channel volumetric input of size (1, 91, 109, 91), and producing a 512-dimensional embedding. Although both encoders share the same architectural design, they are trained with separate weights, so they can capture modality-specific anatomical and microstructural patterns without interference.

**Figure 2:**
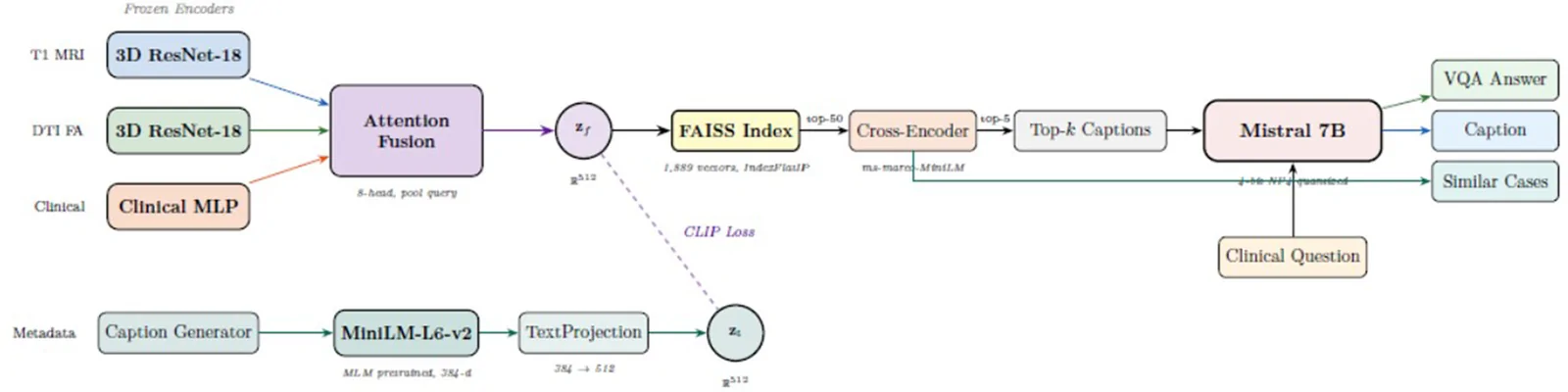
*Model architecture*. Three modality-specific encoders produce ℓ_2_-normalized 512-d embeddings (z_T1_, z_DTI_, z_Clin_). Each embedding passes through a per-modality masking gate ( × m_i_) that randomly drops modalities during training (T1w: 10%, DTI-FA: 30%, Clinical: 5%). The masked embeddings are fused *via* 8-head cross-attention with a learnable pool query, producing z_f_ ∈ R^512^ that feeds six MLP task heads. Dashed lines indicate Stage 1 pairwise contrastive (CLIP) pre-training.

Clinical features are processed by a multi-layer perceptron (MLP) encoder. Four continuous neuropsychological scores are provided as inputs: ADAS-11, ADAS-13, MMSE, and MoCA, together with a learnable embedding for APOE genotype (a categorical risk factor with two alleles). With this design, the model can incorporate both quantitative cognitive measures and genetic risk information into a unified representation.

A critical design decision is the strict exclusion of target variables (diagnosis, sex, age, and amyloid status) from the clinical input space. This prevents label leakage, which would lead to artificially inflated performance (e.g., near-perfect accuracy due to direct label encoding). By omitting these variables, the model is forced to learn meaningful relationships among imaging features and clinical indicators.

Each modality encoder produces a 512-dimensional embedding, and all embeddings are L2-normalized prior to fusion. This normalization ensures compatibility with cosine similarity metrics used in contrastive training and enforces balanced contributions across modalities.

#### 3.2.2 Missing-modality handling via attention fusion

Handling missing modalities is a central challenge for real-world applications of any decision support tool. MEMOIR-VLM addresses this using a cross-attention-based fusion mechanism augmented with explicit modality masking. Each modality embedding is passed through a binary masking gate (m_i_ ε {0,1}), which determines whether the modality is present or dropped. During training, these gates are sampled stochastically according to modality-specific dropout probabilities (T1w: 10%, DTI-FA: 30%, clinical: 5%), reflecting empirical availability patterns in the dataset. At inference time, gating is deterministic and directly reflects the modalities available for a given subject. The fusion module employs multi-head cross-attention with a learnable pooling query that attends over the available modality embeddings. As attention operates on a variable-length set of modality tokens, the model can adapt to any subset of inputs without requiring imputation or separate models. The output is a single fused representation *Z*_f_ ε *R*^512^, which encodes the combined information from all available modalities.

#### 3.2.3 Multi-task prediction heads

The fused representation is passed to five parallel prediction heads, each implemented as a multi-layer perceptron with architecture 512 → 256 → 128 → K, where K is the output dimensionality of specific heads; that is, the number of classes for classification tasks or number of regression outputs; and intermediate dropout regularization. These heads jointly predict a diverse set of clinical targets, including diagnosis (both multi-class and binary), sex, age, and CDR-SB.

This multi-task learning formulation encourages the shared representation to capture complementary aspects of disease progression, spanning structural brain changes and cognitive decline. By jointly optimizing across tasks, the model benefits from inductive transfer, improving robustness and generalization.

### 3.3 Training procedure

#### 3.3.1 Two-stage training protocol

Training proceeds in two stages to decouple representation learning from task-specific optimization. In Stage 1, contrastive pretraining is performed using a CLIP-style InfoNCE loss (Oord et al., 2018; Radford et al., 2021), aligning embeddings across modalities by maximizing similarity between representations of the same subject while minimizing similarity across different subjects. Loss is computed only for modality pairs that are simultaneously available, ensuring compatibility with missing-modality scenarios. Training uses a batch size of 16, as unlike large-scale vision-language CLIP models that require very large batches (32k+) to generate diverse negatives across millions of image-text pairs, our contrastive objective operates over ~3 modality pairs per subject (T1↔DTI, T1↔Clinical, DTI↔Clinical), so the number of informative negatives scales with the number of subjects rather than pair diversity. Given the training set size of 1,889 subjects, a batch size of 16 provides sufficient negative diversity without redundancy. Training was performed on a single NVIDIA V100 (32 GB GPU), with peak VRAM of approximately 16–22 GB under AMP (FP16), dominated by activations for the 91 × 109 × 91 volumetric inputs.

In Stage 2B, the model is fine-tuned for downstream prediction tasks using a multi-task objective. Differential learning rates are applied, with a smaller learning rate for the encoder backbone and a larger one for task heads, stabilizing training while enabling prediction layers to rapidly converge. The optimal model is selected early in training (epoch 5), beyond which models begin to overfit. Table 5 summarizes the training hyperparameters for the multimodal encoder.

**Table 5:** Training hyperparameters for the multimodal encoder.

Hyperparameter	Value
Batch size	16
Optimizer	AdamW (weight decay = 0.01)
Stage 1 learning rate	1 × 10^4^
Stage 2B backbone LR	1 × 10^5^
Stage 2B task head LR	5 × 10^4^
LR scheduler	Cosine annealing
Gradient clipping	1.0
Mixed precision	AMP (FP16)
Label smoothing	0.1
Stage 1 epochs (contrastive)	30
Stage 2B epochs (multi-task)	30 (best checkpoint: epoch 5)

#### 3.3.2 Loss functions and task weights

The overall loss function is a weighted sum of task-specific objectives. Classification tasks use focal loss (γ = 2) or cross-entropy with class weighting to address class imbalance, while regression tasks are optimized using Smooth L1 loss after normalization to [0,1]. Table 6 describes the task-specific loss functions and weights for Stage 2B multi-task training.

**Table 6:** Task-specific loss functions and weights for Stage 2B multi-task training.

Task	Loss function	Weight	Notes
Diagnosis 3-class	Focal Loss (γ = 2)	3.0	Primary task; class-weighted
Diagnosis Binary (CN vs Dementia)	Focal Loss (γ = 2)	1.0	Extreme-class separation
Sex	Cross-entropy	1.0	Class-weighted
Age	Smooth L1 (normalized)	0.5	Normalized to [0, 1]
CDR-SB	Smooth L1 (normalized)	0.5	Normalized to [0, 1]

#### 3.3.3 Modality dropout strategy

To ensure robustness to incomplete inputs, modality dropout is applied during training. Each modality is independently dropped according to its empirical absence rate, exposing the model to all possible modality combinations. This forces the encoder to learn redundant and complementary representations, ensuring reliable performance under partial data conditions. Table 7 describes the per-modality dropout probabilities during training.

**Table 7:** Per-modality dropout probabilities during training.

Modality	Drop probability (%)	Rationale
T1w MRI	10	Available for all subjects; low masking rate
DTI-FA map	30	Available for ~40% of subjects; higher masking
Clinical scores	5	Near-universal availability; minimal masking

### 3.4 Retrieval-augmented VQA

The second stage of MEMOIR-VLM extends the frozen encoder with a retrieval-augmented language generation pipeline, enabling interpretability and natural-language interaction.

#### 3.4.1 Pipeline overview

The retrieval-augmented pipeline (Figure 3) operates in 5 stages: embedding generation, similarity-based retrieval, re-ranking, prompt construction, and LLM-based generation. The fused embedding *Z*_f_ is the central representation linking imaging data to textual reasoning. The embedding stage includes the frozen multi-modal encoders which produce a 512-D fused embedding z_f_ from brain scans and clinical features. The retrieval stage includes a FAISS IndexFlatIP (Johnson et al., 2019) which retrieves the “top-50” most similar training subjects by cosine similarity of fused embeddings. This re-ranking stage uses a *ms-marco-MiniLM* cross-encoder (Hugging Face, 2025) which pairwise-scores top-20 candidates on (query caption, candidate caption) pairs and returns top-5 candidates. The prompting stage has top-5 candidate captions concatenated and inserted into a structured Mistral 7B (Mistral AI, 2023) prompt template (diagnosis, or captioning). The generation stage has a Mistral 7B Instruct (4-bit NF4) which generates a structured natural-language answer and the response is parsed by a two-tier regex/keyword parser.

**Figure 3:**
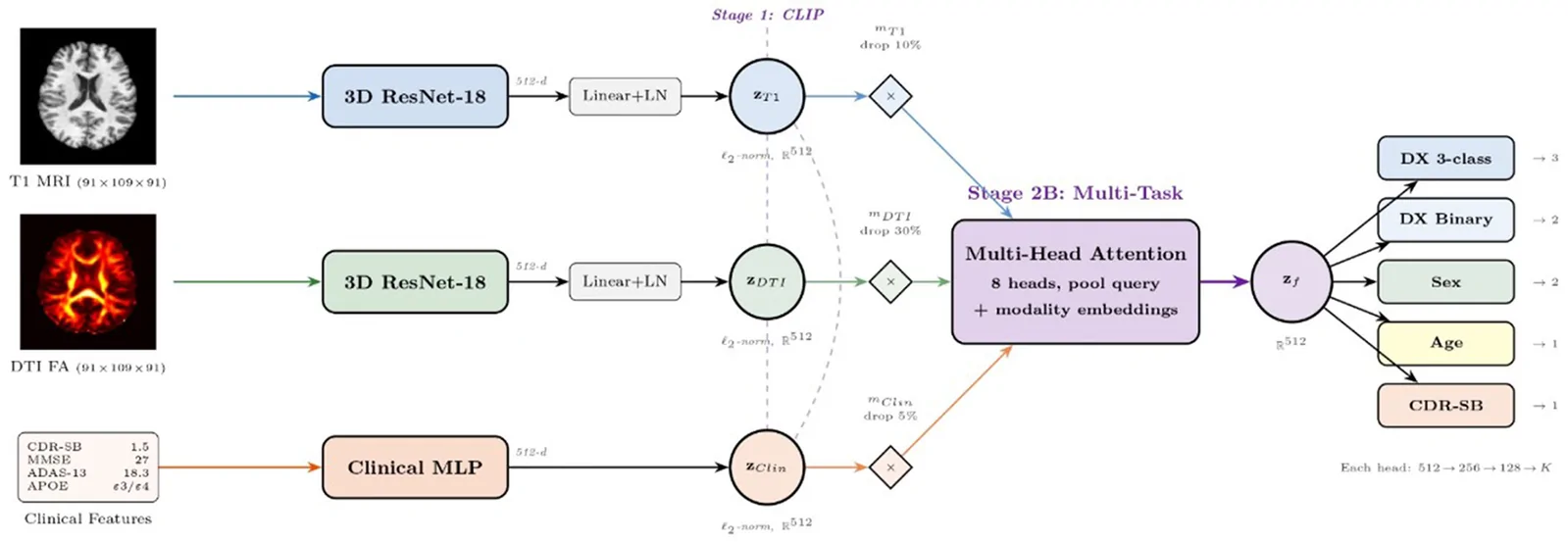
*End-to-end VQA pipeline***. Left:** Frozen multi-modal encoders produce fused embeddings, z_f_ ∈ R^512^. **Center:** FAISS retrieves the top-50 similar training subjects, cross-encoder reranks to top-5. **Bottom:** Text encoder (contrastive-aligned with z_f_) enables cross-modal retrieval. **Right:** Mistral 7B generates answers from retrieved context. Three outputs: retrieval (similar cases), captioning (clinical descriptions), and VQA (question answering).

#### 3.4.2 Text encoder training

A lightweight text encoder is introduced to bridge the gap between imaging modalities and language. The base model, all-MiniLM-L6-v2 (22.7M parameters, 384-dimensional output), undergoes two-stage training. In Stage 1, masked language modeling (MLM) is performed on a domain-specific corpus of 26,889 clinical sentences (1,889 real subject captions supplemented by 25,000 synthetically generated profiles (with the structure explained below) spanning realistic age, diagnostic class, and clinical score distributions). Four clinical domain tokens are added to the vocabulary: Alzheimer's, cognitively, dementia, and genotype. Synthetic augmentation ensures coverage of diverse clinical scenarios and reduces overfitting. For each subject, the caption includes: age, sex, diagnosis, clinical scores (MMSE, CDR-SB, ADAS-11, ADAS-13, MoCA when available), APOE genotype, and imaging modalities present. An example caption would appear as follows:

“*82-year-old male subject diagnosed as Cognitively Normal (CN). Clinical scores: MMSE*=*29.0*,

*CDR-SB*=*0.5, ADAS-11* = *2.7, ADAS-13* = *4.7. APOE genotype: e3/e3. Imaging: T1 MRI available.”*

In Stage 2, the MLM-pretrained backbone is frozen and a lightweight projection layer (384 → 512, with LayerNorm, GELU, and Dropout; 462K trainable parameters) is trained with symmetric InfoNCE loss (temperature τ = 0.07) to align text embeddings with the frozen fused image embeddings z_f_. This projection-only fine-tuning ensures that text and fused embeddings occupy overlapping regions of the same 512-dimensional space, enabling cross-modal similarity search. This alignment enables cross-modal retrieval and allows textual queries to access imaging representations in the shared embedding space. Table 8 summarizes both training stages.

**Table 8:** Text encoder training configuration for both pre-training stages.

Parameter	Stage 1: MLM	Stage 2: Contrastive
Training corpus	26,889 sentences (25K synthetic + 1,889 real)	1,889 real subject pairs
Epochs	30	50
Batch size	32	64
Learning rate	5 × 10^5^	1 × 10^4^ (cosine)
Loss function	Masked Language Modeling (35% mask)	Symmetric InfoNCE (τ = 0.07)
Trainable parameters	22.7M (full backbone)	462K (projection layer only)

#### 3.4.3 FAISS retrieval and reranking

Two FAISS IndexFlatIP indices are built from the 1,889 training subjects; one over fused embeddings (z_f_) and one over projected text embeddings. Both use L2-normalized vectors with exact inner-product search, ensuring no approximation error for the dataset size. For each query, FAISS retrieves the top-50 most similar training subjects by cosine similarity; then a cross-encoder (ms-marco-MiniLM-L-6-v2) re-scores the top-20 candidates on the basis of pairwise caption similarity, and the top-5 most relevant subjects are forwarded to the LLM. This two-stage retrieval approach balances computational efficiency with semantic precision, to provide the LLM with high-quality contextual information.

#### 3.4.4 LLM-based generation and prompt engineering

Mistral-7B-Instruct-v0.3 serves as the primary generation backbone, loaded in 4-bit NF4 quantization (BitsAndBytes), reducing VRAM requirements from approximately 14 GB (FP16) to approximately 4 GB while maintaining generation quality. Two specialized prompt templates are employed, each wrapped in the Mistral instruction format ([INST]...[/INST]):

*Diagnosis prompt:* A constrained prompt that requires the model to produce output in the exact format “Diagnosis: < CN|MCI|Dementia>” followed by a one-sentence rationale, preventing verbose multi-class outputs that confuse downstream parsers;

*Captioning prompt:* An open-ended prompt requesting a free-form clinical description covering likely diagnosis and confidence, inferred clinical characteristics, and notable patterns (e.g., age group).

The exact prompt templates are described in the Supplementary material. A two-tier answer parser extracts structured predictions from LLM responses: a primary regex searches for the structured format “Diagnosis: CN/MCI/Dementia”, and a keyword-first-occurrence fallback is applied for outputs that deviate from the template. The search order is randomized to avoid class-position bias.

It is important to note that the LLM never receives raw brain image data. The “visual” understanding in MEMOIR-VLM is grounded entirely in the frozen encoder's embedding space: brain scans are converted to 512-d vectors by the ResNet-18 branches, FAISS retrieves training subjects in that embedding space, and only the text captions of those retrieved subjects are passed to the LLM as context. This ensures interpretability and aligns with case-based reasoning paradigms in clinical practice.

#### 3.4.5 Caption settings

Because the captions of retrieved training subjects are generated from subject metadata, they contain a diagnosis field. This creates two potential routes by which the ground-truth label can reach the answer without any diagnostic inference: the cross-encoder may rerank candidates by matching the diagnosis string in the query caption against that in the candidate captions, and the language model may simply copy the stated majority label from its context. To quantify these effects, we evaluate the VQA layer under two caption settings on the same model. In the labeled setting, retrieved captions state the neighbour's diagnosis. In the masked setting, deployment-realistic because a query subject's diagnosis is by definition unknown at inference, the diagnosis field is withheld from all captions. FAISS retrieval is identical across the two settings: the same fused query embedding, index, and top-50 candidate set; so the only quantity that varies is the caption text read by the reranker and the language model. Both settings are additionally compared against a retrieval-only k-NN majority vote over the retrieved neighbours' labels, with no language model in the loop.

### 3.5 Experiments

#### 3.5.1 Modality ablation

To quantify the specific contribution of each input modality, a systematic ablation study was designed to evaluate all seven possible modality combinations using the best-checkpoint multimodal encoder. Non-selected modalities are masked at inference time by setting their attention gate to zero, with no modification of model weights. The seven configurations span both clinical-inclusive combinations (four variants) and imaging-only combinations (three variants), described in Table 9. All seven configurations were evaluated on the full 474-subject held-out test set across all six prediction tasks.

**Table 9:** Modality ablation configurations evaluated at inference time.

Configuration	T1w	DTI-FA	Clinical	Purpose
T1 + DTI + Clinical (Full)	✓	✓	✓	Full-modality baseline
T1 + Clinical	✓	✗	✓	Assess DTI contribution
DTI + Clinical	✗	✓	✓	Assess T1 contribution
Clinical only	✗	✗	✓	Clinical scores as sole input
T1 + DTI	✓	✓	✗	Imaging-only regime
T1 only	✓	✗	✗	Minimum imaging baseline
DTI only	✗	✓	✗	White-matter-only baseline

#### 3.5.2 Subset and imaging-only experiments

As DTI-FA is not always available (39.4% of the full test set), a dedicated subset analysis was performed to compare model performance across three subject groups: (A) the 120 dual-modality subjects (T1 + DTI) tested under all four clinical-inclusive masking configurations; (B) the 354 T1w-only subjects, compared under T1w+Clinical vs. Clinical-only masking; and (C) all 474 subjects assessed under the natural (all-available-modality) vs. Clinical-only configurations to quantify the marginal benefit of imaging. This stratified design isolates DTI-specific effects from the confounding influence of clinical covariates.

A separate encoder was trained from scratch using only T1w MRI and DTI FA inputs; clinical features were masked to zero during both training and evaluation. This experiment measures the true predictive capacity of brain structure in isolation, unconfounded by clinical score correlations, and yields a lower bound on the imaging contribution to each task.

CDR-SB was used in some cases as a clinical input and in other cases as a regression target. To avoid using clinical variables highly correlated with diagnosis, we also retrained the model in the case where CDR-SB was removed from the inputs and retained as a prediction target only. The clinical input set was now ADAS-11, ADAS-13, MMSE, MoCA, and APOE genotype. We report the comparison between results as a comparison between original values and the new corrected model in Table 10.

**Table 10:** Comparison results between original model and CDR-SB removed as input model.

Task (metric)	Corrected	Original (leaky)
DX3 balanced accuracy	0.682	0.707
DX3 macro-F1	0.681	0.703
DX2 balanced accuracy	0.913	0.933
Sex accuracy	0.555	0.575
Age MAE (years)	5.96	6.31
CDR-SB MAE	1.11	0.97^†^

^†^This result was obtained from the original model in which CDR-SB was included as an input, resulting in target leakage.

#### 3.5.3 VQA modality and LLM ablations

The VQA pipeline is further evaluated under three query-time modality configurations: full modality (T1 + DTI + Clinical), also termed standard conditions, imaging-only (T1 + DTI, no clinical), and T1-only. In all cases, the FAISS index remains unchanged (built from full-modality training embeddings); only the query embedding at inference is masked. This design isolates the effect of missing clinical features on retrieval quality and subsequent LLM generation accuracy, mirroring realistic deployment scenarios such as population screening where cognitive assessments may not be available when images are acquired.

Three LLM backbones are evaluated as drop-in replacements for the generation stage, with all other pipeline components held constant (Table 11). Mistral 7B Instruct v0.3 serves as the primary backbone; Gemma 4 26B MoE (a larger Mixture-of-Experts model) (Google, 2026) and MedGemma 1.5 4B IT (a medical-domain fine-tuned vision-language model) (Sellergren et al., 2026) are evaluated as comparators. All three models are loaded in 4-bit NF4 quantization. As retrieval is performed entirely before LLM generation, retrieval accuracy is identical across all three conditions; any differences in VQA accuracy are therefore attributable solely to the generation step. This controlled comparison investigates whether model scale, domain specialization, or instruction-following ability is the dominant factor for structured clinical VQA.

**Table 11:** LLM backbone specifications for ablation experiment.

Property	Mistral 7B	Gemma 4 26B MoE	MedGemma 1.5 4B
Architecture	Dense transformer	Mixture-of-Experts	Dense (vision-language)
Total parameters	7B	26B (~4B active)	4B
Domain	General	General	Medical
Quantization	4-bit NF4	4-bit NF4	4-bit NF4
GPU memory	~4.5 GB	~10.9 GB	~2.5 GB
Context window	32,768 tokens	128,000 tokens	—

#### 3.5.4 Text generation evaluation

Beyond classification accuracy, the quality of generated text is assessed using four complementary metrics, widely used in natural language processing studies, on 150 stratified test subjects (50 per diagnostic class). Reference strings are structured ground-truth captions generated from subject metadata (age, sex, diagnosis, clinical scores, and APOE). Four metrics are computed:

*BLEU:* n-gram precision with smoothing, measuring exact lexical overlap (Papineni et al., 2002);

*ROUGE-L:* longest common subsequence F1, capturing sentence-level structural similarity (Lin, 2004);

*BERTScore:* token-level cosine similarity using RoBERTa-large contextual embeddings, capturing semantic fidelity (Zhang et al., 2019); and

*SBERT cosine similarity:* sentence-level embedding similarity using all-MiniLM-L6-v2, measuring holistic semantic proximity (Reimers and Gurevych, 2019).

Two generation tasks are compared under this evaluation: open-ended captioning and structured VQA summarization. The top-10 retrieved subjects (rather than the top-5 used for classification) are used as LLM context in this experiment, providing a richer basis for long-form generation. These metrics assess lexical accuracy and semantic fidelity of generated clinical descriptions.

#### 3.5.5 Statistical analysis of the imaging contribution

To assess whether the imaging contribution to diagnosis is statistically reliable, we computed confidence intervals on paired differences in performance between modality configurations. Because the model was trained once, we did not perform a cross-seed paired test; instead we report, as the primary analysis, stratified paired-bootstrap 95% confidence intervals obtained by resampling the held-out test set. For each comparison, test subjects are resampled with replacement, stratified by true diagnostic class so that balanced accuracy remains well-defined (10,000 iterations, fixed seed); both configurations are scored on the same resampled subjects, and the difference is recorded. We report the point difference and its 95% percentile interval. As a formal hypothesis test, we also report McNemar's exact test on the 2 × 2 table of per-subject correct/incorrect outcomes, with discordant counts (b, c) and exact *p*. A two-sided bootstrap *p*-value is reported as a supporting quantity only.

We evaluate 5 comparisons on each of the 3-class (DX3, *n* = 474) and binary (DX2, CN vs. Dementia, *n* = 311) tasks: (A) Clinical-only vs. DTI-FA+Clinical; (B) Clinical-only vs. Full; (C) Clinical-only vs. T1w+Clinical; (D) Full vs. a two-layer clinical MLP baseline; and (E) Full vs. an AutoGluon tabular baseline. Comparison A is pre-specified and reported unadjusted; comparisons B–E are secondary and receive Holm–Bonferroni adjustment within each metric. Because DTI-FA is available for only 179 of 474 test subjects, and the modality mask only ever removes modalities, the DTI-FA+Clinical and Clinical-only configurations yield identical predictions for the 295 subjects without DTI; we therefore report comparison A both on the full test set and restricted to the 179 DTI-available subjects, where the effect is undiluted. For age regression we compare imaging-only (T1w+DTI-FA) against clinical-only using per-subject absolute errors, with a paired-bootstrap 95% CI on the mean-absolute-error difference (primary) and Wilcoxon signed-rank and paired *t*-tests (secondary).

### 3.6 Implementation details

All encoder training was performed on a single NVIDIA Tesla V100 32 GB GPU. The full offline embedding computation for 2,363 subjects requires approximately 20 min; MLM pre-training of the text encoder completes in 15 min; contrastive alignment takes 5 min; and full VQA evaluation on 150 subjects requires approximately 45 min. FAISS index construction from 1,889 training embeddings completed in under one second on a CPU. For single-patient clinical deployment, the pipeline required 0.5 s to compute a fused embedding from preprocessed neuroimaging volumes, less than 1 ms for FAISS retrieval, 0.5 s for cross-encoder reranking, and 3–5 s for LLM generation, yielding an end-to-end latency of 4–6 s per subject on a single V100 GPU. Total VRAM requirement is approximately 8 GB when the encoder and Mistral 7B are loaded simultaneously, making the system deployable on a single consumer-grade GPU, making it suitable for real-world deployment. The modular design enables efficient offline indexing and fast query-time inference, supporting both batch analysis and interactive clinical use. All experiments are implemented in PyTorch with the Hugging Face Transformers and PEFT libraries. FAISS operations use the *faiss-cpu* package. All pretrained model weights are publicly available on the Hugging Face Model Hub.

## 4 Results

This section reports the outcomes of all experiments described in **Section 3**, following a structured progression from core multimodal encoder performance to retrieval-augmented reasoning and generation. Specifically, we present: (i) overall encoder performance across classification and regression tasks, (ii) detailed per-class diagnostic behavior and prediction calibration, (iii) modality ablation analyses across all input combinations, (iv) targeted subset analyses isolating the contribution of DTI and structural MRI, (v) an imaging-only training baseline, and (vi) evaluation of the retrieval-augmented VQA pipeline, including LLM ablation and generation quality. Across all experiments, *bold* values indicate the best-performing configuration within each row.

### 4.1 Overall multimodal encoder performance

Table 12 reports the performance of the best model checkpoint (Stage 2B, epoch 5) on the full 474-subject held-out test set for all five prediction tasks, with CDR-SB in training data. Results are separated into classification (balanced accuracy, macro F1, AUC) and regression (MAE) tasks. In the corrected model, from which CDR-SB is excluded as an input (Section 3.5.2), the model achieves a balanced accuracy of 68.2% (macro-F1 = 0.681) for three-class diagnosis classification (CN/MCI/Dementia). Binary classification of cognitively normal vs. dementia subjects reached 91.3% balanced accuracy, showing that the learned representation strongly separates the extremes of the disease spectrum. Age regression yields a mean absolute error of 5.96 years. CDR-SB regression MAE is 1.11 points across the subjects with valid scores; this is higher than the 0.97 obtained by the original model, an expected consequence of removing CDR-SB from the input rather than a regression in model quality, since the original figure benefited from the target being partially present among the inputs. This is close to the inter-rater reliability of the test (Dauphinot, 2024; McDougall et al., 2021; Rockwood et al., 2000). Sex prediction was the weakest classification task at 0.575 balanced accuracy (AUC 0.597); reflecting limited sex-discriminating signals in the combination of structural MRI, DTI, and cognitive scores used; a finding consistent across all modality ablation configurations.

### 4.2 Three-way diagnosis: per-class analysis and prediction calibration

Detailed per-class analysis reveals important insights into model behavior. As shown in Tables 13a, b, dementia is the easiest class to identify, achieving 86% recall and F1-score of 0.82, followed by CN (recall 71%). MCI was the most challenging class, with a recall of only 55%. This difficulty reflects the intrinsic heterogeneity of MCI, which lies between normal cognition and dementia and exhibits overlapping structural and clinical characteristics with both. A substantial fraction of MCI subjects are misclassified as either CN (24.5%) or Dementia (20.2%), consistent with its transitional nature. Even so, extreme misclassifications are nearly absent: only 3 out of 474 subjects (0.6%) are confused between CN and Dementia. This indicates that the model learns a well-ordered representation of disease progression, where errors are almost exclusively between adjacent stages. Prediction calibration further supports this. The model assigns very low Dementia probability to CN subjects (~8%) and *vice versa*, indicating strong separation at the extremes.

### 4.3 Modality ablation study

Tables 14 and 15 report performance across all seven modality combinations for clinical-inclusive and imaging-only configurations respectively, on the full 474-subject test set. Non-selected modalities are masked at inference time with no change in model weights. Several patterns emerge consistently across tasks. First, clinical scores are the dominant modality. Clinical-only 3-class diagnosis classification balanced accuracy (0.700) approaches the full-modality result (0.707), confirming that the three cognitive and biomarker scores (CDR-SB, ADAS-11/13, MMSE) carry the majority of diagnostic signals. Second, DTI provides incremental value over T1 MRI alone for 3-way diagnosis, and age estimation. The DTI+Clinical combination achieves the highest AUC for DX3 (0.873), and the lowest CDR-SB MAE (0.91) among all clinical-inclusive configurations. DTI+Clinical also achieves the best age MAE in the imaging-only group (6.22 years for the 2-modality group). Within the model's own ablation, adding DTI-FA to the clinical scores raises three-class balanced accuracy (0.666 → 0.701); however, this within-architecture gain does not exceed a simple clinical-feature baseline (Section 4.10), so we do not claim it as evidence that imaging improves diagnosis. The one task for which imaging yields an unambiguous and absolute gain is age regression, where the imaging-only configuration (MAE 6.10 years) outperforms the clinical-only configuration (6.57 years).

**Table 12a:** Overall classification performance—best model (Stage 2B, epoch 5).

Task	Balanced accuracy	Macro F1	AUC-ROC	*N* (no. of test scans)
DX 3-class (CN/MCI/ Dementia)	**0.707**	0.703	0.865	474
DX binary (CN vs. Dementia)	**0.933**	0.932	0.981	311
Sex (Male/Female)	0.575	0.563	0.597	474

Bold values indicate the best-performing configuration within each row.

**Table 12b:** Regression task performance.

Task	MAE	Mean predicted	Mean true	*N* (no. of test scans)
Age (years)	**6.31 years**	78.0 yr	75.5 yr	474
CDR-SB	**0.97**	2.72	2.48	419

Bold values indicate the best-performing configuration within each row.

**Table 13a:** DX 3-class confusion matrix (full test set, *n* = 474, all modalities).

True / Predicted	CN	MCI	Dementia
CN (*n* = 168)	**119 (70.8%)**	48 (28.6%)	1 (0.6%)
MCI (*n* = 163)	40 (24.5%)	**90 (55.2%)**	33 (20.2%)
Dementia (*n* = 143)	2 (1.4%)	18 (12.6%)	**123 (86.0%)**

Bold values indicate the best-performing configuration within each row.

**Table 13b:** DX 3-class per-class classification report.

Class	Precision	Recall	F1-score	Support
CN	0.74	0.71	0.72	168
MCI	0.58	0.55	0.56	163
Dementia	**0.78**	**0.86**	**0.82**	143

Bold values indicate the best-performing configuration within each row.

**Table 14a:** Modality ablation results for classification tasks (clinical combinations).

Task	T1w + DTI-FA+Cl.			T1w + Cl.			DTI-FA + Cl.			Clinical Only		
	BA	F1	AUC	BA	F1	AUC	BA	F1	AUC	BA	F1	AUC
DX3	**0.707**	**0.703**	0.865	0.692	0.688	0.863	0.701	0.688	**0.873**	0.700	0.680	**0.873**
DX2	0.933	0.932	**0.981**	0.932	0.932	**0.981**	**0.938**	**0.941**	0.978	**0.938**	**0.941**	0.976
Sex	0.575	0.563	0.597	0.574	0.560	**0.599**	0.583	0.583	0.579	**0.590**	**0.589**	0.574

Best per row in bold. Cl. stands for Clinical Features. BA stands for Balanced Accuracy, F1 stands for F1 Score and AUC stands for AUC-ROC. DX3 stands for 3 class Diagnosis Classification (CN, MCI, and Dementia), and DX2 stands for CN vs Dementia Classification.

**Table 14b:** Modality ablation results for classification tasks (imaging only combinations).

Task	T1w + DTI-FA			T1w Only			DTI-FA Only		
	BA	F1	AUC	BA	F1	AUC	BA	F1	AUC
DX3	**0.598**	**0.585**	**0.773**	0.587	0.570	0.771	0.388	0.337	0.572
DX2	**0.848**	**0.839**	**0.935**	0.833	0.823	**0.935**	0.528	0.455	0.651
Sex	**0.554**	**0.515**	0.614	0.523	0.462	**0.625**	0.524	0.457	0.537

Best per row in bold. Cl. stands for Clinical Features. BA stands for Balanced Accuracy, F1 stands for F1 Score and AUC stands for AUC-ROC. DX3 stands for 3 class Diagnosis Classification (CN, MCI and Dementia), and DX2 stands for CN vs. Dementia Classification.

**Table 15:** Modality ablation results for regression tasks (lower MAE is better).

Modality	Task	
	Age MAE	CDR-SB MAE
T1w + DTI-FA + Cl.	6.31	0.97
T1w + Cl.	6.34	0.95
DTI-FA + Cl.	6.49	**0.91**
Clinical Only	6.57	0.92
T1w + DTI-FA	**6.10**	1.73
T1w Only	6.17	1.74
DTI-FA Only	6.46	2.25

Best results are in bold. Cl. stands for Clinical Features.

The modality ablation is the central analysis for the question of imaging value, and its interpretation changes materially once the CDR-SB input leak is removed. In the original (leaky) model, clinical-only three-class balanced accuracy (0.700) was indistinguishable from the full-modality result (0.707), and no meaningful imaging contribution could be demonstrated. In the corrected model, clinical-only DX3 falls to 0.666, and the addition of imaging improves it to 0.701 for DTI-FA + Clinical. This improvement is statistically supported [paired difference +0.034, 95% CI (+0.009, +0.060); McNemar *p* = 0.012; Section 4.11], and is larger on the subset of subjects for whom diffusion imaging is actually available [+0.071, 95% CI (+0.002, +0.141)]. DTI-FA alone sits near the 3-class chance line (0.392), confirming that imaging is not independently sufficient for staging. Taken together, and with the reliability analysis of Section 4.11, these results indicate that imaging and cognitive assessment are complementary rather than redundant on three-class diagnosis: imaging contributes a statistically measurable signal when integrated alongside cognitive context, but not in isolation. On the binary task the imaging contribution does not reach significance (Section 4.11), consistent with CN and dementia being well separated by cognition alone.

### 4.4 VQA pipeline: retrieval performance

Prior to LLM generation, the FAISS retrieval stage identifies the most similar training subjects from the 1,889-subject index. Table 16a reports overall retrieval accuracy for both fused and text embedding spaces, measured at several values of k using the standard top-k retrieval metric: retrieval accuracy at @k denotes the fraction of test subjects for whom the majority diagnosis among the k most similar retrieved neighbors matches the true label, with @1 being the strictest case where only the single top-ranked neighbor is considered. Table 16b disaggregates fused embedding retrieval by diagnostic class. Text embedding retrieval achieves near-perfect accuracy (96.2% at @1) because the clinical captions explicitly encode diagnostic labels, rendering text-to-text matching near-trivial. The more meaningful metric is fused embedding retrieval (69%), which operates on learned imaging and clinical representations without explicit label information. Per-class retrieval reveals that people with dementia are easiest to retrieve (72.0% at @5), CN subjects are intermediate (73.8% at @5), and MCI subjects are hardest (57.1% at @5)—consistent with MCI's heterogeneous embedding distribution in the 512-dimensional fused space. The retrieval accuracies reported here are computed purely in the fused embedding space: the top-k neighbors are retrieved by cosine similarity of the 512-d fused embeddings and their majority diagnosis is taken, with no caption text and no cross-encoder involved. This is distinct from any value computed after cross-encoder reranking over captions, which, because the captions contain a diagnosis field, is subject to label exposure. The fused-embedding majority@5 of 0.675 is consistent with the per-class values of Table 16b; it should not be confused with the label-containing reranked value discussed below.

### 4.5 VQA pipeline: diagnosis accuracy

Table 17 compares three generation strategies on 150 stratified test subjects (50 per class)—retrieval-only majority voting, full VQA (Mistral 7B with structured prompt), and a hybrid approach that uses retrieval when top-5 agreement is ≥3/5 and falls back to LLM otherwise. Under labeled captions the Mistral 7B VQA pipeline reaches 94.7% overall diagnostic accuracy; however, as we show below, this figure reflects ground-truth diagnoses present in the retrieved captions and does not hold once those labels are masked. In the deployment-realistic masked setting, no language model exceeds the retrieval-only k-NN majority baseline of 0.673, which in turn matches the encoder's own three-class accuracy of 0.682. We therefore treat 94.7% as a label-exposure artifact, decomposed below, rather than as a diagnostic result. CN and Dementia reach 94 and 92% accuracy respectively. Confusion analysis confirms that no configuration confuses CN with Dementia or vice versa and all errors are adjacent-class (CN → MCI or Dementia → MCI), indicating that the VQA pipeline preserves the ordinal structure of Alzheimer's progression from the retrieved context. Examples of results from the model are included in the Supplementary material. We distinguish two retrieval baselines: the fused-embedding k-NN majority (no caption access) retrieves neighbors purely by embedding similarity and takes their majority label; on the 150-subject VQA set this yields 0.673, consistent with the 0.675 reported over the full test set in Table 16a. The reranked retrieval majority additionally passes candidates through the cross-encoder, which scores captions containing the diagnosis field; this raises the majority to 0.927 purely through caption-label access, and is not a deployable baseline. The 0.927 figure reported as “retrieval majority vote (top-5)” in Table 17 was of this second, label-containing kind, which is why it can not be reconciled with the embedding-space per-class values of Table 16b.

**Table 16a:** FAISS retrieval accuracy by majority vote across *k* values (all 474 test subjects).

Configuration	@1	@3	@5	@10
Fused embeddings	0.688	**0.696**	0.675	0.690
Text embeddings	**0.962**	**0.958**	**0.962**	**0.958**

Bold values indicate the best-performing configuration within each row.

**Table 16b:** Per-class fused embedding retrieval accuracy (majority@k, all 474 test subjects).

Diagnosis class	N	@1	@5	@10
CN	168	0.726	0.738	**0.780**
MCI	163	0.626	0.571	0.577
Dementia	143	0.713	**0.720**	0.713

Bold values indicate the best-performing configuration within each row.

**Table 17:** VQA diagnosis accuracy on 150 stratified test subjects (50 per class).

Method	CN	MCI	Dementia	Overall
Retrieval majority vote (top-5)	-	-	-	0.927
VQA—Mistral 7B	**0.940**	**0.980**	**0.920**	**0.947**
Hybrid (retrieval + LLM)	0.900	**0.980**	0.900	0.927

Best results are in bold.

The retrieval-augmented layer does not add diagnostic accuracy once caption labels are hidden. In the masked setting, no language model exceeds the retrieval-only k-NN majority baseline of 0.673, and that baseline itself merely matches the encoder's own three-class accuracy (0.682). Retrieval does not beat the encoder, and the language model does not beat retrieval. The strongest backbone under masking, Gemma 4 26B (0.653), only approaches the baseline; Mistral 7B (0.473) and MedGemma 1.5 4B (0.413) fall substantially below it, both over-calling impairment—masked Mistral predicts CN for only 1 of 150 subjects. In the labeled setting, the accuracy reaches 0.94. Because retrieval is identical across the two caption settings, the difference between them isolates the effect of caption-mediated label exposure. Two distinct leaks are separable. First, with the diagnosis present in the captions, the cross-encoder matched the query caption's diagnosis text against the candidates' and returned diagnosis-homogeneous neighbors, so that even the retrieval-only majority rose to 0.907. S, the language model then copied those stated labels. Removing the diagnosis from the captions collapses both: retrieval falls to 0.673, matching the encoder, and the language model to 0.41–0.65, below it. The original accuracy therefore reflected label exposure rather than diagnostic skill. This also inverts the conclusion of our language-model ablation. Under labeled captions, Mistral 7B appeared the strongest backbone; under masked captions it is among the weakest, and Gemma 4 26B is strongest. The original ranking thus measured fidelity in copying labels stated in context, not clinical reasoning ability.

Because retrieval is identical across caption settings, comparing the retrieval-majority baseline between them isolates the effect of caption-label access. Masking the diagnosis costs 23 percentage points on the three-class task but only 1.5 on the binary task. CN and Dementia are well separated in the fused embedding (encoder DX2 = 0.913), so retrieval recovers correct-class neighbors from embedding similarity without the diagnosis text; MCI is not separable in that space, so on the three-class task the reranker relied on the leaked label to assemble diagnosis-homogeneous neighborhoods. The label leak operated specifically where the encoder was weak.

The class-wise pattern of results provides an independent signature of the caption-label leak. Under labeled captions, MCI was the best VQA class (0.98) despite being the worst class for both the encoder (recall 0.55) and fused-embedding retrieval (0.571 @5, Table 14b). This arises because MCI is the least separable class in the embedding space, so fused-embedding retrieval cannot assemble a homogeneous MCI neighborhood, but the cross-encoder, scoring captions that state each neighbour's diagnosis, used the diagnosis text to assemble one. The reranker's label access thus operated most strongly on precisely the class the encoder could not resolve. Under the masked-caption protocol the reversal disappears and MCI VQA accuracy returns to a level consistent with the encoder and with embedding-space retrieval, confirming that the labeled-caption MCI result reflected label copying rather than diagnostic skill. This is corroborated by the task-level decomposition: masking costs the retrieval-majority baseline 23 points on the three-class task but only 1.5 on the well-separated binary task, the entire three-class effect being concentrated on the MCI boundary.

### 4.6 VQA modality ablation

Table 18 reports VQA performance under three query-time modality configurations. The FAISS index remains unchanged (built from full-modality training embeddings) across all conditions; only the query embedding is masked at inference time. Removing clinical features from the query embedding causes FAISS retrieval accuracy to drop by 16.2 percentage points at @5 (0.730 → 0.568), confirming that the fused embedding space is dominated by clinical information. These VQA accuracies (0.947 → 0.860 across query configurations) are measured under labeled captions and therefore inherit the caption-label exposure quantified in Section 4.5; they should not be read as evidence that the language model compensates for weaker retrieval. Under the deployment-realistic masked-caption setting, VQA does not exceed the retrieval-only baseline in any configuration. What the query-time modality ablation does establish, independently of the leak, is that retrieval quality in the fused embedding space degrades gracefully as modalities are removed (FAISS @5 0.730 → 0.568), consistent with the encoder's own graceful degradation under modality dropout. CN accuracy degrades most (0.940 → 0.760 for T1w-only), consistent with cognitively healthy subjects having structurally healthy brains that overlap with early MCI in embedding space when clinical anchors are absent. The addition of DTI-FA to T1w provides only marginal retrieval benefit when clinical features are removed (T1w+DTI-FA @5: 0.568; T1w-only @5: 0.576), confirming that the added value of DTI-FA depends on being integrated alongside clinical context.

**Table 18:** VQA modality ablation: retrieval accuracy, and diagnosis accuracy, across three query configurations (*n* = 474 for retrieval; n = 150 for VQA diagnosis; *n* = 100 for amyloid).

Configuration	Standard (T1w + DTI-FA + Cl.)	Imaging only (T1w + DTI-FA)	T1w only
FAISS Retrieval @5	**0.730**	0.568	0.576
VQA Diagnosis Acc.	**0.947**	0.860	0.847
– CN	**0.940**	0.800	0.760
– MCI	**0.980**	**0.960**	**0.960**
– Dementia	**0.920**	**0.820**	**0.820**

Cl. stands for Clinical Features.

Acc. stands for Accuracy. Bold values indicate the best-performing configuration within each row.

### 4.7 LLM backbone comparison

Table 19 presents the 3-way comparison of Mistral 7B Instruct v0.3, Gemma 4 26B MoE, and MedGemma 1.5 4B IT across all diagnostic VQA configurations and diagnostic tasks. Retrieval accuracy is identical across all three LLMs, as it is determined entirely by the FAISS stage prior to generation. Under labeled captions, Mistral 7B outperforms both comparators on all diagnostic VQA configurations. As shown in Section 4.5, this ranking inverts under masked captions—where Gemma 4 26B is strongest and Mistral 7B among the weakest—indicating that the labeled-caption ranking measures fidelity in copying stated labels rather than diagnostic reasoning. We therefore do not present Mistral 7B as the preferred backbone on diagnostic grounds. Its advantages are in format adherence (≈100% parseable outputs vs. 66.7% for MedGemma) and in text-generation quality, together with a low resource footprint (approximately 4.5 GB VRAM, 4–6 s per inference), which make it the preferred backbone for the natural-language interface stage of MEMOIR-VLM.

**Table 19:** Three-way LLM backbone comparison across all VQA configurations.

Metric	Mistral 7B	Gemma 4 26B MoE	MedGemma 1.5 4B
DX VQA—Standard	**0.947**	0.927	0.507
DX VQA—Imaging Only	**0.860**	0.793	0.487
DX VQA—T1w Only	**0.847**	0.773	0.480
CN Accuracy—Standard	**0.940**	0.900	0.480
CN Accuracy—T1w Only	**0.760**	0.580	0.400
MCI Accuracy—Standard	**0.980**	**0.980**	0.500
Dementia Accuracy—Standard	**0.920**	0.900	0.540
Format adherence	**≈100%**	**≈100%**	66.7%
GPU memory	≈4.5 GB	≈10.9 GB	≈2.5 GB

Best results are in bold. ↑ indicates higher is better. Standard indicates T1w + DTI-FA + Clinical Features, whereas Imaging Only indicates T1w + DTI-FA.

### 4.8 Text generation quality

Table 20 reports automatic text generation quality metrics for the Mistral 7B backbone evaluated on 150 stratified test subjects using the top-10 retrieved context. Results are presented for open-ended captioning and structured VQA summarisation, broken down by diagnostic class.

**Table 20:** Text generation quality metrics (Mistral 7B, top-10 context, 150 subjects, 50 per class).

Task/Class	BLEU	ROUGE-L	BERTScore	SBERT CosSim
Captioning				
Overall	0.044 ± 0.025	0.211 ± 0.057	0.866 ± 0.017	0.767 ± 0.077
CN	0.045	0.214	**0.871**	**0.789**
MCI	**0.064**	**0.223**	0.868	0.774
Dementia	0.022	0.197	0.858	0.738
VQA				
Overall	**0.066** **±0.038**	**0.345** **±0.084**	**0.894** **±0.022**	**0.811** **±0.076**
CN	**0.077**	**0.377**	**0.902**	**0.831**
MCI	**0.087**	0.330	0.887	0.787
Dementia	0.035	0.328	0.892	0.815

Values shown as mean ± SD for overall rows; class rows show mean only. BERTScore uses RoBERTa-large contextual embeddings; SBERT uses all-MiniLM-L6-v2. Bold values indicate the best-performing configuration within each row.

BLEU scores are low (0.044 for captioning; 0.066 for VQA), which is expected as the reference strings are structured clinical templates (eg. “82-year-old male diagnosed with CN. MMSE=29.0…”) while Mistral generates free-form prose (“Based on similar subjects, the query subject appears to be…”), and BLEU penalises non-literal n-gram mismatch heavily. Semantic similarity metrics are much more positive. BERTScore reaches 0.894 for VQA (0.866 for captioning), indicating high token-level semantic overlap between generated and reference text; the model seems to capture the correct clinical facts despite expressing them in different surface forms. SBERT cosine similarity of 0.811 for VQA (0.767 for captioning) suggests that generated and reference texts occupy neighboring regions of the sentence embedding space. VQA summarization consistently outperforms open-ended captioning across all four metrics and the three diagnostic classes. This reflects the structured VQA prompt's directive to enumerate diagnosis, demographics, clinical scores, and APOEs; an instruction that steers the LLM toward the same categories as the reference template and produces more numerically precise outputs. The dementia class scores lowest on BLEU and ROUGE-L in captioning (0.022 and 0.197), because Mistral generates more hedging and explanatory language for complex cases, reducing lexical overlap with the reference template.

### 4.9 External validation: zero-shot performance on OASIS-3

The model's zero-shot performance on OASIS-3 against the internal ADNI reference is as follows: binary CN-vs.-impaired diagnosis transfers well (balanced accuracy 0.787, AUC 0.889), notwithstanding the complete absence of the DTI modality and the change of sites, scanners and acquisition protocols. Three-class accuracy degrades to 0.524, driven chiefly by the MCI class, which is small in OASIS-3. Age regression does not transfer reliably (MAE 8.43 years vs. 5.96 internally), as OASIS-3 skews younger than ADNI's training range. CDR-SB MAE is nominally lower externally (0.90 vs. 1.11), but this reflects the cohort's composition rather than improved transfer: OASIS-3 is predominantly cognitively normal (751/1048), so CDR-SB ≈ 0 constitutes an easy regime.

### 4.10 External baselines

To situate the model relative to simpler and alternative approaches, we evaluate four baselines on the identical subject-level split. A two-layer MLP (128, 64) and an AutoGluon tabular predictor are trained on the same clinical features the model's clinical encoder consumes; a text-only language model (Llama-3.2-3B) receives those features as text, with no images and no retrieval; and MedGemma 1.5 4B, a medical vision-language model, is evaluated within the pipeline (Section 4.7).

### 4.11 Statistical reliability of the imaging contribution

Table 21 reports all ten diagnostic comparisons; Figure 4 shows the corresponding paired-difference intervals. On three-class diagnosis, only comparison A; the addition of DTI-FA to the clinical inputs; has a 95% confidence interval that excludes zero: the difference is +0.034 [95% CI (+0.009, +0.060); McNemar *p* = 0.012]. Restricted to the 179 subjects with diffusion imaging available, where the effect is not diluted by identical-prediction subjects, the improvement roughly doubles to +0.071 [95% CI (+0.002, +0.141)], and the interval still excludes zero. By construction, the McNemar discordant counts are identical for the full set and the DTI-available subset (*b* = 12, *c* = 29; *p* = 0.012), since concordant identical-prediction subjects do not enter the test.

**Table 21a:** Paired difference in balanced accuracy (config B—config A), full test set.

Comparison	A Bal Acc	B Bal Acc	Difference (95% CI)	Excl. 0	McNemar *p*	Holm *p*
Three-class diagnosis (DX3), *n* = 474						
A Clin vs. DTI+Clin	0.666	0.701	+0.034 [+0.009, +0.060]	Yes	0.012^*^	-
B Clin vs. Full	0.666	0.682	+0.016 [−0.031, +0.062]	No	0.498	1.00
C Clin vs. T1+Clin	0.666	0.683	+0.016 [−0.028, +0.062]	No	0.488	1.00
D Full vs. MLP	0.682	0.705	+0.023 [−0.023, +0.068]	No	0.379	1.00
E Full vs. AutoGluon	0.682	0.699	+0.016 [−0.031, +0.064]	No	0.549	1.00
Binary diagnosis (DX2, CN vs. Dementia), *n* = 311						
A Clin vs. DTI+Clin	0.893	0.910	+0.017 [−0.007, +0.041]	No	0.210	-
B Clin vs. Full	0.893	0.913	+0.021 [−0.016, +0.058]	No	0.256	1.00
C Clin vs. T1+Clin	0.893	0.906	+0.013 [−0.025, +0.051]	No	0.430	1.00
D Full vs. MLP	0.913	0.924	+0.011 [−0.021, +0.043]	No	0.442	1.00
E Full vs. AutoGluon	0.913	0.919	+0.005 [−0.025, +0.037]	No	0.690	1.00

Positive favors the second configuration. Comparison A is the pre-specified primary result (unadjusted); B–E carry Holm-adjusted McNemar p within each metric.

**Table 21b:** Comparison A (clinical-only vs. DTI-FA + clinical), full set vs. DTI-available subset.

Metric	Sample	*n*	Difference (95% CI)	Excl. 0	McNemar (*b,c*;p)
DX3	Full test set	474	+0.034 [+0.009, +0.060]	Yes	(12, 29); 0.012
DX3	DTI-available only	179	+0.071 [+0.002, +0.141]	Yes	(12, 29); 0.012
DX2	Full (CN/Dem)	311	+0.017 [−0.007, +0.041]	No	(5, 11); 0.210
DX2	DTI-available (CN/Dem)	126	+0.010 [−0.049, +0.063]	No	(5, 11); 0.210

McNemar (b, c) are identical by construction.

DX3 stands for 3 class Diagnosis Classification (CN, MCI and Dementia), and DX2 stands for CN vs. Dementia Classification.

**Figure 4:**
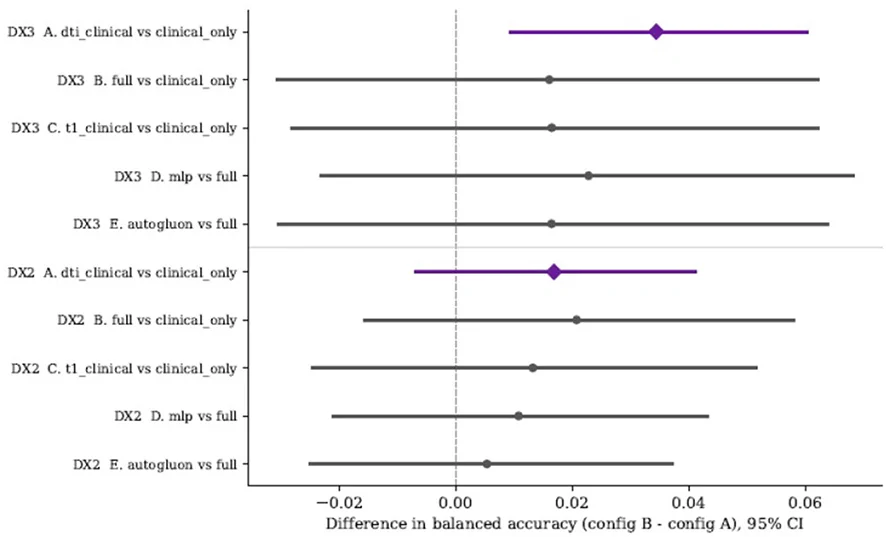
Paired-difference 95% confidence intervals for all ten diagnostic comparisons. The diamond marks comparison A (Clinical-only vs. DTI-FA+Clinical). Only the three-class comparison A lies entirely to the right of zero; all other intervals cross the dashed zero line. Intervals reflect test-set resampling (10,000 stratified paired-bootstrap iterations) and not training-seed variability.

On the binary CN-vs-Dementia task, comparison A does not reach significance [+0.017; 95% CI (−0.007, +0.041); McNemar *p* = 0.210], consistent with the extremes of the disease spectrum being well separated by cognitive scores alone. Every other comparison on both tasks (Clinical-only vs. Full, Clinical-only vs. T1w+Clinical, and both tabular-baseline comparisons) has a confidence interval containing zero. In particular, the two-layer clinical MLP is not statistically distinguishable from the full multimodal model on either task [DX3 difference +0.023, 95% CI (−0.023, +0.068); DX2 difference +0.011, 95% CI (−0.021, +0.043)].

For age regression, the imaging-only configuration achieves lower mean absolute error than the clinical-only configuration by 0.63 years [95% CI (+0.14, +1.11); Wilcoxon *p* = 0.005; paired *t*-test *p* = 0.011], an interval that excludes zero. Imaging therefore provides a statistically supported improvement for age estimation, a target for which cognitive scores are by construction uninformative.

These intervals quantify variability over the held-out test subjects; as the model was trained once, they do not capture training-seed variability, and a confidence interval that contains zero indicates that the effect is not distinguishable from zero given this test set and this trained model.

## 5 Discussion

Our design and testing of MEMOIR-VLM demonstrates that it can perform missing-modality-aware multimodal encoding, multi-task clinical prediction, and retrieval-augmented natural-language reasoning. Unlike many prior AI methods with applications to AD, this overall toolkit offers a range of capabilities and could even be incorporated into a larger agentic AI system.

The three-class diagnosis result (68.2% balanced accuracy in the corrected model) reflects the genuine complexity of Alzheimer's staging. The high binary CN vs. Dementia separation (91.3%) confirms that the multi-task training procedure instills a structured embedding space that respects the ordinal progression of the disease. MCI is difficult to classify, with errors distributed symmetrically toward both diagnostic neighbors, which is natural as MCI is a transitional and heterogeneous state. The modality ablation and subset analyses clarify the complementary roles of each input stream. Clinical scores dominate diagnosis classification as a 0.7 percentage point gap between full-modality and clinical-only 3-way diagnostic performance confirms that the neuropsychological battery encodes the majority of the useful staging information. In clinical practice, cognitive assessment is the primary basis for AD diagnosis. MEMOIR-VLM's diagnostic value lies in its consistent, weighted integration of multiple scores rather than in extracting novel imaging signals for staging *per se*. Imaging, by contrast, is the modality that matters most for tasks where cognitive performance is an unreliable guide—such as biological age estimation. We limit our claim of imaging value to the tasks where it is observed and robust. For diagnosis, imaging does not add accuracy over a simple clinical-feature model (Section 4.11), and we make no incremental-value claim there. For age estimation, imaging is the modality that matters. The imaging-only configuration outperforms the clinical-only configuration by 0.63 years MAE [95% CI (+0.14, +1.11); Wilcoxon *p* = 0.005; Section 4.11], a statistically supported difference consistent with cognitive scores being uninformative about brain structure. These results underscore the importance of systematic ablation rather than assuming that additional modalities always improve performance, particularly when modality availability is non-randomly distributed across the dataset.

Biological sex is ordinarily easy to decode from structural brain MRI (typically >90%), so we read the near-chance balanced accuracy we obtain (0.575) as evidence that the imaging encoders are under-trained on this cohort. This is consistent with two other observations reported here: the fused clinical pathway extracts less signal than a standalone MLP on the same features (Section 4.11), and the DTI+Clinical configuration marginally exceeds the full model on three-class diagnosis. Several design choices additionally suppress the cues sex prediction relies on: L2-normalization of the modality embeddings discards magnitude, and thus the head-size cue that is among the strongest structural correlates of sex; and sex carries the lowest task weight among five heads sharing a diagnosis-oriented embedding. These factors do not fully excuse the result, and we take the near-chance sex accuracy as bounding the representational quality of the current imaging pathway. Whether a higher-capacity or better-trained imaging encoder would recover strong sex decoding and, with it, a genuine imaging gain on diagnosis is an open and likely linked question for future work.

The modality ablation invites a direct question: if cognitive scores alone approach full-modality diagnostic performance, what is the imaging encoder for? Following removal of the CDR-SB input leak (Section 4.3), the gap widens as clinical-only three-class balanced accuracy is 0.666, and adding DTI-FA raises it to 0.701, while DTI-FA alone sits near chance (0.392) indicating that imaging and cognition are complementary rather than redundant. Beyond this aggregate gain, we identify three specific scenarios in which imaging provides clinically meaningful value over cognitive assessment alone. First, imaging is not incremental but necessary wherever cognitive assessment is unavailable or unreliable. A neuropsychological battery requires a trained administrator, sustained patient cooperation, and language- and education-appropriate norms; it is frequently unavailable in emergency neuroimaging, population screening, primary care, and low- and middle-income settings, and it is confounded in patients with delirium, aphasia, depression, low educational attainment, or limited proficiency in the testing language. In these contexts a clinical-only model is not merely less accurate but inapplicable. MEMOIR-VLM's imaging-only configurations retain meaningful performance with the clinical encoder masked entirely (T1w + DTI-FA: DX3 0.58, DX2 0.84), obtained from a scan that has typically already been acquired at no additional patient burden. Second, imaging is the modality that matters for targets on which cognitive performance is an unreliable guide. For age regression, the imaging-only configuration (6.10 years MAE) outperforms the clinical-only configuration (6.57 years), reversing the ordering observed for diagnosis. Age estimation is the elementary case of a broader family of structurally defined targets such as brain-age gap, atrophy quantification, white-matter burden, longitudinal structural change, for which cognitive scores are by construction uninformative. Third, within imaging, DTI-FA contributes over T1w specifically when integrated with clinical context: DTI-FA + Clinical achieves both the best corrected three-class balanced accuracy (0.701) and the lowest CDR-SB MAE (0.91) among clinical-inclusive configurations, consistent with white-matter microstructural change being detectable earlier than macrostructural volumetric loss and therefore informative about severity gradation. That DTI-FA alone is near chance while DTI-FA + Clinical is the strongest configuration reflects FA's character as a sensitive but non-specific marker requiring cognitive anchoring to become diagnostically actionable. We do not claim that MEMOIR-VLM extracts staging information that a well-administered cognitive battery does not already carry. Where a full neuropsychological assessment is available, cognitive testing remains the primary basis for AD diagnosis, and the model's contribution is the consistent, weighted integration of multiple scores rather than the discovery of novel imaging biomarkers for staging *per se*. The clinically meaningful value of the imaging encoder lies in robustness when cognition is absent, in targets cognition cannot address, and in severity gradation.

Our model training strategy uses stochastic modality dropout, which proved effective as performance degraded gradually as modalities are removed rather than catastrophically. The imaging-only model retains meaningful diagnostic accuracy without any clinical inputs. This robustness is clinically relevant for deployment in settings where neuropsychological assessment is unavailable, such as emergency neuroimaging, population screening, or resource-limited environments. The VQA modality ablation reveals an additional resilience property: despite a 16.2 percentage point drop in retrieval accuracy when clinical features are removed, overall VQA diagnosis accuracy falls by only 8.7 points, because the Mistral 7B generation stage partially compensates for weaker retrieval by reasoning over the diagnostic signal present even in imperfectly matched neighbors. This gap between retrieval and VQA degradation is an advantage of the RAG design vs. a pure embedding-space retrieval system. A second key property of the VQA pipeline; in contrast to the multimodal encoder, which produced 3 cross-spectrum confusions (0.6%) in its discriminative classification head (Section 4.2); is that all VQA classification errors are strictly adjacent-class as no modality configuration or LLM backbone ever predicts CN for a true Dementia subject or vice versa. This ordinal error structure is an emergent consequence of the retrieval mechanism as subjects who are grossly dissimilar on the diagnostic spectrum are also dissimilar in embedding space and therefore do not tend to appear in each other's retrieved neighborhoods. From a clinical safety standpoint, the structural prevention of cross-spectrum confusion is highly desirable, and may distinguish MEMOIR-VLM from unconstrained generative systems.

The three-way LLM comparison establishes that instruction-following capacity at scale is the dominant factor for structured clinical VQA, outweighing model size and medical domain specialization. Mistral 7B's superiority over Gemma 4 26B MoE on all diagnosis configurations despite having four times fewer total parameters reflects the strict output format that our diagnostic prompt requires. The choice of LLM backbone for clinical deployment should prioritize instruction-following reliability over domain pretraining. A more flexible answer parser may substantially recover MedGemma's latent performance. The text generation evaluation confirms that MEMOIR-VLM produces semantically faithful clinical descriptions beyond simple classification labels. Low BLEU scores (0.044–0.066) are expected given the surface mismatch between free-form generated prose and structured reference templates. The semantically sensitive metrics are more informative as BERTScore of 0.894 and SBERT cosine similarity of 0.811 for VQA summarisation confirm token-level and sentence-level fidelity to the clinical reference content. The multi-task learning framework produces an embedding space that underpins the entire VQA pipeline. Training simultaneously on five heterogeneous targets encourages the cross-attention fusion module to encode clinically multidimensional similarity into a single 512-d representation, so that FAISS retrieval returns neighbors who resemble the query subject across diagnosis, cognitive severity, and age simultaneously. A set of single-task specialist models would likely achieve higher per-task accuracy, but at the cost of five separate embedding spaces and the loss of this unified similarity structure. The modest 0.7 percentage point 3-way diagnosis penalty (attributable to multi-task training) is a small price for the retrieval coherence and interpretability that the shared representation enables.

Because the retrieval-augmented language model does not exceed a k-NN majority vote once caption labels are masked, we do not claim visual question answering as a diagnostic classifier. Its value is as an interpretable, natural-language interface: given a query embedding, it retrieves comparable reference subjects and produces a readable clinical summary grounded in them, with a traceable evidence chain of the five nearest cases and their full clinical profiles. That capability is clinically useful but it is an explanation capability, not a diagnostic one. The diagnostic pipeline proper is the encoder (DX3 0.682, DX2 0.913), optionally supplemented by retrieval-based k-NN voting for the label (0.673). We report the original figure and its decomposition because the mechanism of a metadata field leaking into a retrieval context is a failure mode likely to recur in retrieval-augmented clinical systems generally, and is worth documenting.

External validation on OASIS-3 provides the clearest evidence that the missing-modality mechanism is functionally load-bearing. The cohort has no processed diffusion data at all, yet the identical ADNI-trained weights run on it zero-shot, with the DTI branch masked, and retain binary diagnostic performance of 0.787 balanced accuracy (AUC 0.889). A model requiring complete modality sets, or relying on generative imputation of the absent modality, could not be applied in this setting without additional machinery and additional hallucination risk. That three-class performance degrades (0.524) while binary performance holds is itself informative: the transferable signal lies at the extremes of the disease spectrum, where structural and cognitive divergence is largest, while the MCI boundary does not transfer.

A plain MLP on the clinical features is statistically indistinguishable from the full multimodal model on both diagnostic tasks: the paired differences favor the MLP only nominally [DX3 +0.023, 95% CI (−0.023, +0.068); DX2 +0.011, 95% CI (−0.021, +0.043)], with both intervals including zero (Section 4.11). A simple tabular model and the multimodal model are not distinguishable on diagnosis on this cohort, where the diagnostic label is largely a function of the cognitive scores—not that the tabular baseline outperforms the multimodal model. What the multimodal architecture adds beyond the tabular baseline is not higher diagnostic accuracy on complete-data subjects but operation under any subset of available modalities, statistically supported gains on 3-class diagnosis and on age estimation, and zero-shot transfer to an external cohort with a modality entirely absent (Section 4.9).What our architecture provides, and what the tabular baselines cannot, is operation under any subset of available modalities and zero-shot transfer to an external cohort with a modality entirely absent (Section 4.9); that capability, together with the leakage audit, is where we locate the contribution of this work. Whether a better-trained clinical encoder or fusion could recover a genuine imaging gain remains an open question.

Several concrete pathways emerge for future clinical translation. The binary CN vs. Dementia separation (encoder balanced accuracy 91.3%) and its zero-shot transfer to OASIS-3 (0.787 balanced accuracy, AUC 0.889) suggest that, if further validated on cohorts beyond ADNI, the MEMOIR-VLM encoder could serve as a scalable first-stage screening component to flag individuals requiring specialist referral, helping to address the severe global shortage of dementia care specialists documented in low- and middle-income countries. The imaging-only encoder configurations retain meaningful diagnostic performance with the clinical encoder masked entirely (T1w + DTI-FA: DX2 0.84), supporting use in resource-limited settings where neuropsychological assessment may not be routinely available. We rest these deployment arguments on the encoder and its external transfer rather than on VQA accuracy, which does not exceed the encoder once caption labels are masked. The retrieval output of the five most similar historical subjects with their full clinical profiles provides a form of case-based reasoning directly interpretable by clinicians, offering a transparent audit trail consistent with regulatory and medicolegal standards for AI-assisted decision support.

For a clinical decision support tool, the relevant question is not only whether a prediction is correct but whether a clinician can inspect and contest the basis for it. MEMOIR-VLM provides two mechanisms for this. The first is case-based: retrieval returns the five most similar documented subjects with their full clinical profiles, so that a prediction can be interrogated as “this patient most closely resembles these five previously characterized cases”. This mirrors clinical reasoning from comparable prior cases, produces an auditable evidence chain rather than an opaque probability, and is a property of the architecture rather than a *post-hoc* reconstruction of it. The second is the modality ablation of Section 4.3, which quantifies at the level of each input stream what that stream contributes to each prediction target. We regard the modality-level question of how much did imaging contribute at all, relative to cognition, as logically prior to the voxel-level question, and as the one a clinician most needs answered before any spatial attribution map can be responsibly interpreted.

Our natural-language VQA interface enables open-ended clinical querying without requiring additional model calls or specialized infrastructure, supporting a collaborative rather than prescriptive model of AI integration. Finally, the 4–6 s per-patient end-to-end inference latency on a single consumer GPU (~8 GB VRAM) shows that clinical deployment would not require high-performance computing. Beyond Alzheimer's disease, the architectural principles of MEMOIR-VLM may be applicable to the challenges of radiologic AI more generally, and may also have applications in brain-computer interface design (BCI). BCI systems routinely integrate heterogeneous neural signals such as EEG, fMRI, structural MRI, and behavioral data, but with variable availability across subjects and sessions. This is precisely the problem that the missing-modality cross-attention fusion mechanism was designed to address. The convergence of multimodal encoders, LLMs, and retrieval-augmented generation demonstrated here represents a promising blueprint for the next generation of interpretable, neuroimaging AI.

## 6 Limitations and future directions

All experiments were conducted on ADNI, a North American research cohort recruited under standardized acquisition protocols. Cognitive reserve, APOE allele frequencies, and rates of comorbid pathology differ substantially with ancestry, and scanner hardware, field strength, and acquisition parameters vary widely in clinical practice compared to the controlled multisite ADNI environment. Generalizability to diverse real-world clinical populations cannot be assumed, and future validation on ancestrally and geographically diverse external cohorts would be needed prior to deployment. Future work should investigate domain adaptation and federated learning (Rieke et al., 2020; Stripelis et al., 2021, 2024) to extend MEMOIR-VLM to multi-institution settings without requiring centralized data sharing. A related open challenge is continual learning (Bruno et al., 2025; Lee and Lee, 2020; Yi et al., 2023), as new cohorts and acquisition protocols become available, the model should ideally be updated incrementally rather than retrained from scratch. However, continual fine-tuning of LLMs can cause catastrophic forgetting (Alammar et al., 2024; Gupta et al., 2021; Perkonigg et al., 2021), where performance on previously learned distributions degrades as the model adapts to new data. FDA approval of radiologic AI systems does not cover cases where the model is updated and performance can drift over time. Developing continual learning strategies that allow MEMOIR-VLM to incorporate new institutional datasets and emerging biomarker modalities while preserving previously acquired knowledge represents an important and largely open area of research for clinically deployed neuroimaging AI.

Even after removing CDR-SB from the input set, the remaining cognitive variables (MMSE, ADAS-11, ADAS-13, MoCA) remain correlated with the diagnostic label. So, whenever these scores are provided as inputs, the framework has access to proxy diagnostic information and does not operate free of it. In ADNI, as in clinical practice, the diagnostic label is itself derived in part from cognitive assessment, so any model given cognitive scores has partial access to the target. We therefore do not claim that MEMOIR-VLM extracts diagnostic signals that cognitive assessment does not already carry. What we claim is narrower, and is supported by the corrected modality ablation and its reliability analysis (Section 4.11): imaging adds a statistically measurable signal on top of cognition for 3-class diagnosis [clinical-only 0.666 vs. 0.701 with DTI-FA added; +0.034, 95% CI (+0.009, +0.060)]. The imaging-only configurations, in which the clinical encoder is masked entirely at inference, provide a lower bound on what brain structure alone supports at prediction time (DX3 0.58; DX2 0.84 for T1w + DTI-FA). This is an imaging-only-input bound rather than an imaging-only-supervision result. The shared representation and the supervision labels were still shaped during training by the cognitive scores and by a diagnostic label that is itself partly cognition-derived. A proxy-free evaluation would require supervision from a signal independent of cognitive assessment. This is the most important direction for establishing the true standalone value of the imaging pathway, and it should take two complementary forms in future work: (i) imaging-only supervision, in which the encoder is trained and evaluated without any cognition-derived target; and (ii) validation against independent biomarkers such as amyloid or tau PET, plasma biomarkers, or imaging-derived labels, which supply ground truth that cognitive scores cannot proxy. The amyloid-prediction extension outlined below is a concrete instance of the second. Supervising diagnosis-adjacent targets on PET-derived labels, with cognitive-derived variables excluded, would break the proxy dependence that all cognition-input configurations retain. Two further observations reinforce that the current imaging pathway is not the source of the framework's diagnostic performance. Near-chance sex prediction (0.555 in the corrected model), on a task that is normally easy from structural MRI, indicates that the imaging encoders are not fully learning the anatomical features that the modality affords. Together with the modest imaging-only diagnostic bound, the model's present contribution lies in its missing-modality operation and cross-site transfer rather than in the representational strength of the imaging pathway and it is why proxy-free, imaging-supervised validation is needed to determine whether a better-trained imaging encoder would carry genuine standalone diagnostic signal.

In any real deployment the query subject's diagnosis is unknown, and the reference captions must therefore be constructed without a diagnosis field. Under this condition the retrieval-augmented layer performs at the level of the encoder rather than above it: no language model exceeds a retrieval-only k-NN majority vote (0.673), which in turn matches the encoder's own three-class accuracy (0.682). The deployment configuration we recommend is therefore one where the diagnostic label is produced by the multimodal encoder, optionally with k-NN voting, while the retrieval-augmented language layer supplies the natural-language rationale, the retrieved comparator cases, and the auditable evidence chain. Constructing the reference index from label-free captions is a design requirement of that configuration, not an implementation detail. Under masked captions the language models over-call impairment substantially—masked Mistral 7B predicts CN for only 1 of 150 ADNI subjects; so calibration or an abstention mechanism would be a prerequisite for clinical use even of the generated summaries.

We validated the framework on an external cohort, but OASIS-3, like ADNI, is a North American research cohort recruited under standardized protocols. Zero-shot transfer to OASIS-3 therefore establishes cross-site, cross-scanner, cross-protocol generalization, and because DTI is entirely absent there, the practical viability of the missing-modality design. It does not establish generalisability to ancestrally and geographically diverse clinical populations. Cognitive reserve, APOE allele frequencies, and rates of comorbid pathology differ substantially with ancestry, and clinical acquisition conditions vary far more widely than either research cohort reflects. Validation on ancestrally diverse and non-Western cohorts, and on data acquired in routine clinical rather than research settings, remains necessary prior to any deployment. The out-of-distribution failure of age regression on OASIS3's younger cohort is a concrete illustration of how quickly a task can cease to transfer when the target distribution shifts. A reasonable next step is to train the core algorithm over datasets with a larger age range.

Voxel-level imaging explainability such as saliency maps, Grad-CAM, occlusion sensitivity, or attention visualization over the image volumes; is beyond the scope of the present study, and we acknowledge this as an explicit limitation. We regard it as a genuine gap and we outline below both why standard attribution methods cannot be applied naively to this architecture and what a principled analysis would require, which we intend to pursue in future work. The difficulty is architectural rather than merely computational: attribution methods such as Grad-CAM and occlusion sensitivity are not straightforwardly transferable to a missing-modality fusion architecture. In a single-modality classifier the attribution target is a fixed input tensor and the gradient path is unambiguous. In MEMOIR-VLM, the prediction derives from a fused embedding whose composition varies per subject: the modality mask gates a different subset of streams for each individual, so that a subject with T1w and clinical data alone, and a subject with all three modalities, are effectively processed by different sub-networks. A saliency map over the T1w volume therefore has no stable cross-subject meaning; it is conditional both on which other modalities were present and on how much attention mass the fusion layer allocated to the T1w stream in that case. Pooling such maps across a cohort in which DTI-FA is available for only 39.4% of subjects would conflate attribution with availability. Furthermore, a voxel map cannot answer the question that most matters clinically such as “how much the imaging pathway contributed relative to cognition” and, presented alone, would render the imaging pathway vividly legible while leaving the dominant clinical pathway invisible, inviting over-interpretation. This risk is a systematic failure mode of common explainability tools in neuroimaging and has been well documented, and we have previously reported substantial divergence between attribution methods on this task (Chattopadhyay et al., 2024a). For these reasons we have addressed attribution at the modality level, through systematic ablation across all seven modality subsets, which quantifies directly what each input stream contributes to each target. A principled voxel-level analysis remains an important direction for future work and would require attribution to be computed conditionally on modality-availability pattern, reported separately for each modality subset rather than pooled, paired with the fusion layer's per-subject modality attention weights so that spatial attribution is always accompanied by a measure of the imaging pathway's contribution, and validated against a reference such as voxel-based morphometry in the shared template space. In the interim, the framework provides two forms of interpretability that do not depend on voxel-level attribution: the modality-level ablation of Section 4.3, which quantifies each input stream's contribution to each target, and the case-based retrieval output, which grounds every prediction in five documented comparator subjects with full clinical profiles.

The statistical reliability analysis of Section 4.11 quantifies variability over the held-out test subjects through stratified paired bootstrapping, but because the model was trained once, these intervals do not capture training-seed variability. A confidence interval that excludes zero establishes that an effect is robust to which subjects were held out; it cannot establish that a re-trained model would reproduce the same effect. Multi-seed training, which would allow a paired test across independent runs, is a natural next step but was beyond the scope of the present study.

A key direction for future work, and the most direct route to the proxy-free validation discussed above, is the extension of MEMOIR-VLM to amyloid prediction as an explicit task supervised on a biomarker independent of cognitive assessment. The current framework does not include amyloid status as a prediction target; future iterations should incorporate a dedicated amyloid training objective with amyloid-specific contrastive pretraining, ensuring that AV45 SUVR is strictly excluded from the clinical input features to avoid label leakage, with amyloid positivity derived solely from PET-based thresholding as a supervision signal. A larger amyloid-labeled training cohort, or the addition of tau PET or plasma biomarkers (Jack Jr et al., 2023; Nakamura et al., 2018), could further strengthen the learned representations for preclinical amyloid screening. Extending the VQA pipeline with an amyloid-specific FAISS index, built from subjects with known amyloid labels, would be a natural next step, potentially enabling the system to serve as a low-cost first-stage filter prior to expensive and invasive confirmatory testing such as amyloid PET imaging or lumbar puncture, though this would require rigorous prospective validation before any clinical deployment.

The cross-encoder reranking stage compares the query subject's caption against the captions of retrieved training neighbors. In the current implementation, training subject captions include the ground-truth diagnostic label, a form of indirect label access that could inflate retrieval quality relative to true clinical deployment, where the query subject's diagnosis is unknown and must therefore be omitted from their caption. While this does not affect the encoder or classification heads, it may overstate the benefit of reranking in the VQA pipeline. Future work should evaluate a masked-label caption variant, in which the query caption is generated without a diagnosis field. This should produce a more conservative and deployment-realistic estimate of cross-encoder performance.

DTI availability in the current dataset is 39.4%. Extending the dataset to a higher DTI coverage fraction through harmonized multi-cohort aggregation and explicitly modeling the missingness mechanism rather than treating it as missing-at-random, would strengthen the scientific validity of the missing-modality findings. Like all retrieval-augmented generation systems, MEMOIR-VLM is susceptible to LLM hallucination (Asgari et al., 2025; Ji et al., 2023) such as the generation of factually unsupported claims about a subject's profile or prognosis. The current pipeline contains no hallucination guardrails beyond the structured prompt format, and all generated text should be treated as assistive rather than authoritative without clinician review. Future iterations should incorporate grounding verification such as checking that specific numerical claims in the generated output are traceable to values in the retrieved captions. Confidence calibration, could also be based on retrieval agreement among top-k neighbors, and this could provide a signal for when the LLM's generation should be flagged for uncertainty. Evaluation of smaller, locally deployable instruction-tuned models and retrieval-grounded generation frameworks would also strengthen the reliability and auditability of the output. The current model operates on a single cross-sectional scan and predicts current clinical status. Alzheimer's disease is fundamentally longitudinal, and clinical questions often concern future trajectory such as whether an MCI subject will convert to dementia, how rapidly a patient's CDR-SB will progress, or whether a cognitively normal individual's amyloid burden will reach the symptomatic threshold within a given timeframe. Extending MEMOIR-VLM to longitudinal prediction, through multi-timepoint embedding or by retrieval based on longitudinal trajectory similarity, is a high-priority direction that should increase the system's value for prognosis and treatment planning.

## 7 Conclusions

We presented MEMOIR-VLM, a framework for Alzheimer's disease characterization that addresses three limitations in much of the neuroimaging AI literature—reliance on complete multimodal data, narrow single-task prediction, and the lack of interpretable natural-language reasoning. By combining a missing-modality-aware cross-attention encoder trained jointly on five clinical prediction targets with a retrieval-augmented VQA pipeline grounded in learned neuroimaging embeddings, the encoder achieves 91.3% binary CN vs. Dementia balanced accuracy, while the retrieval-augmented VQA layer functions as an interpretable natural-language interface rather than an independent diagnostic classifier. The LLM backbone comparison showed that instruction-following capacity at scale outweighs model size and medical domain specialization for structured clinical generation. With an end-to-end inference latency of 4–6 s on a single consumer GPU, MEMOIR-VLM could be deployed without high-performance computing infrastructure, and its case-based retrieval output provides clinicians with transparent, evidence-grounded reasoning rather than opaque probability scores. This work brings together multimodal deep learning, large language models, and retrieval-augmented generation within a principled architecture for clinically translatable neuroimaging AI.

## Data Availability

Publicly available datasets were analyzed in this study. This data can be found here: https://adni.loni.usc.edu.

## References

[B1] Alammar Z. Alzubaidi L. Zhang J. Li Y. Gupta A. Gu Y. (2024). Generalisable deep learning framework to overcome catastrophic forgetting. Intell. Syst. Appli. 23:200415. doi: 10.1016/j.iswa.2024.200415

[B2] Alzheimer's Disease International (2021). Dementia Statistics. Available online at: https://www.alzint.org/about/dementia-facts-figures/dementia-statistics/ (Accessed May 15, 2026).

[B3] Asgari E. Montaña-Brown N. Dubois M. Khalil S. Balloch J. Yeung J. A. . (2025). A framework to assess clinical safety and hallucination rates of LLMs for medical text summarisation. NPJ Digit. Med. 8:274. doi: 10.1038/s41746-025-01670-7 PMC12075489 40360677

[B4] Ayyachamy S. Alex V. Khened M. Krishnamurthi G. (2019). “Medical image retrieval using Resnet-18,” in Medical Imaging 2019: Imaging Informatics for Healthcare, Research, and Applications, Vol. 10954 (SPIE), pp. 233–241.

[B5] Bruno P. Quarta A. Calimeri F. (2025). Continual learning in medicine: a systematic literature review. Neural Process. Lett. 57:2. doi: 10.1007/s11063-024-11709-7

[B6] Camus V. Payoux P. Barré L. Desgranges B. Voisin T. Tauber C. . (2012). Using PET with 18F-AV-45 (florbetapir) to quantify brain amyloid load in a clinical environment. Eur. J. Nucl. Med. Mol. Imaging. 39, 621–31. 10.1007/s00259-011-2021-8 PMC3315642 22252372

[B7] Can D. C. Dang L. D. Tang Q. H. Ly D. M. Ha H. Blanc G. . (2025a). VisTA: vision-text alignment model with contrastive learning using multimodal data for evidence-driven, reliable, and explainable alzheimer's disease diagnosis. arXiv [preprint]. *arXiv:2502.01535*.

[B8] Can D. C. Tang Q. H. Ha H. Nguyen B. T. Chén O. Y. (2025b). “REMEMBER: retrieval-based explainable multimodal evidence-guided modeling for brain evaluation and reasoning in zero-and few-shot neurodegenerative diagnosis,” in Proceedings of the 33rd ACM International Conference on Multimedia (pp. 11179–11188). doi: 10.1145/3746027.3754809

[B9] Chang Y. Wang X. Wang J. Wu Y. Yang L. Zhu K. . (2024). A survey on evaluation of large language models. ACM Trans. Intell. Syst. Technol. 15, 1–45.

[B10] Chattopadhyay T. Jagad C. Kush R. Desai V. D. Thomopoulos S. I. Villalón-Reina J. E. . (2025a). “Evaluating synthetic diffusion MRI maps created with diffusion denoising probabilistic models,” in Medical Imaging 2025: imaging informatics, Vol. 13411 (SPIE), 148–159.

[B11] Chattopadhyay T. Jagad C. Senthilkumar P. Thomopoulos S. I. Villalón-Reina J. E. Thompson P. M. (2025c). “Using diffusion transformers to generate synthetic diffusion scalar maps for data augmentation,” in 2025 21st International symposium on biomedical image processing and analysis (SIPAIM) (IEEE), 1–4.

[B12] Chattopadhyay T. Joshy N. A. Jagad C. Gleave E. J. Thomopoulos S. I. Feng Y. M. . (2024a). “Comparison of explainable AI models for MRI-based Alzheimer's disease classification,” in 2024 20th International symposium on medical information processing and analysis (SIPAIM) (IEEE), 1–4.

[B13] Chattopadhyay T. Joshy N. A. Ozarkar S. S. Buwa K. Feng Y. Laltoo E. . (2024b). “Brain age analysis and dementia classification using convolutional neural networks trained on diffusion MRI: tests in Indian and North American cohorts,” in 2024 46th Annual International Conference of the IEEE Engineering in Medicine and Biology Society (EMBC) (IEEE), pp. 1–7. doi: 10.1109/EMBC53108.2024.10781599 40039079

[B14] Chattopadhyay T. Komandur D. Naik J. Buwa K. Ozarkar S. S. Thomopoulos S. I. . (2023a). “Can structural MRIs be used to detect amyloid positivity using deep learning?” in 2023 19th International symposium on medical information processing and analysis (SIPAIM) (IEEE), 1–4.

[B15] Chattopadhyay T. Mehendale G. Thomopoulos S. I. Joshi H. Venkatasubramanian G. John J. P. . (2025b). “Generating diffusion MRI scalar maps from T1-weighted images using Reversible GANs,” in 2025 21st International symposium on biomedical image processing and analysis (SIPAIM) (IEEE), 1–4.

[B16] Chattopadhyay T. Ozarkar S. S. Buwa K. Joshy N. A. Komandur D. Naik J. . (2024c). Comparison of deep learning architectures for predicting amyloid positivity in Alzheimer's disease, mild cognitive impairment, and healthy aging, from T1-weighted brain structural MRI. Front. Neurosci. 18:1387196. doi: 10.3389/fnins.2024.1387196 PMC11250587 39015378

[B17] Chattopadhyay T. Senthilkumar P. Ankarath R. H. Patterson C. Gleave E. J. Thomopoulos S. I. . (2025d). “Predicting longitudinal cognitive decline via multimodal deep learning fusing brain MRI and clinical data,” in 2025 21st international symposium on biomedical image processing and analysis (SIPAIM) (IEEE), 1–4.

[B18] Chattopadhyay T. Senthilkumar P. Ankarath R. H. Patterson C. Gleave E. J. Thomopoulos S. I. . (2026). Deep learning to predict future cognitive decline: a multimodal approach using brain MRI and clinical data. Front. Neuroimaging 5:1726037. doi: 10.3389/fnimg.2026.1726037 PMC12913073 41717278

[B19] Chattopadhyay T. Singh A. Joshy N. A. Thomopoulos S. I. Nir T. M. Zheng H. . (2023b). “Predicting dementia severity by merging anatomical and diffusion MRI with deep 3D convolutional neural networks,” in 18th International symposium on medical information processing and analysis, Vol. 12567 (SPIE), 90–99.

[B20] Chen Q. Hong Y. (2024). “Medblip: bootstrapping language-image pre-training from 3D medical images and texts,” in Proceedings of the Asian Conference on Computer Vision (pp. 2404–2420). doi: 10.1007/978-981-96-0908-6_6

[B21] Dauphinot V. (2024). Reliability of the assessment of the clinical dementia rating scale from the analysis of medical records in comparison with the reference method. Alzheimer's Res. Therapy 16:198. doi: 10.1186/s13195-024-01567-9 PMC11376013 39238042

[B22] Dhinagar N. J. Jagad C. Senthilkumar P. Thomopoulos S. I. Khan M. H. Liew S. L. (2026b). CALM-VLM: calibration and selective prediction in vision-language models for reliable brain MRI classification. bioRxiv 2026-04. doi: 10.1109/ISBI61048.2026.11515735

[B23] Dhinagar N. J. Ozarkar S. S. Buwa K. U. Thomopoulos S. I. Owens-Walton C. Laltoo E. . (2025a). “Parameter efficient fine-tuning of transformer-based masked autoencoder enhances resource constrained neuroimage analysis,” in Medical imaging 2025: computer-aided diagnosis, Vol. 13407 (SPIE), 271–278.

[B24] Dhinagar N. J. Senthilkumar P. Thomopoulos S. I. Thompson P. M. (2026a). “MAVeRiC-AD: mixture-of-experts agentic vision-language ensemble for robust MRI classification of Alzheimer's disease,” in Medical imaging 2026: computer-aided diagnosis, Vol. 13926 (SPIE), 1392602.

[B25] Dhinagar N. J. Thomopoulos S. I. Laltoo E. Thompson P. M. (2023). “Efficiently training vision transformers on structural MRI scans for Alzheimer's disease detection,” in 2023 45th Annual International Conference of the IEEE Engineering in Medicine and Biology Society (EMBC) (IEEE), pp. 1–6. doi: 10.1109/EMBC40787.2023.10341190 38083552

[B26] Dhinagar N. J. Thomopoulos S. I. Thompson P. M. (2025b). “Leveraging a vision-language model with natural text supervision for MRI retrieval, captioning, classification, and visual question answering,” in 2025 47th Annual International Conference of the IEEE Engineering in Medicine and Biology Society (EMBC) (IEEE), pp. 1–7. doi: 10.1109/EMBC58623.2025.11251809 41336503

[B27] Folstein M. F. Folstein S. E. McHugh P. R. (1975). Mini-mental state. J. Psychiatr. Res. 12, 189–198. doi: 10.1016/0022-3956(75)90026-6 1202204

[B28] Gao Y. Xiong Y. Gao X. Jia K. Pan J. Bi Y. . (2023). Retrieval-augmented generation for large language models: a survey. arXiv [preprint]. arXiv:2312.10997.

[B29] GBD 2019 Dementia Forecasting Collaborators (2022). Estimation of the global prevalence of dementia in 2019 and forecasted prevalence in 2050: an analysis for the global burden of disease study 2019. Lancet Public Health 7, e105–e125. doi: 10.1016/S2468-2667(21)00249-8 PMC8810394 34998485

[B30] Golovanevsky M. Eickhoff C. Singh R. (2022). Multimodal attention-based deep learning for Alzheimer's disease diagnosis. J. Am. Med. Informat. Assoc. 29, 2014–2022. doi: 10.1093/jamia/ocac168 PMC9667156 36149257

[B31] Google (2026). gemma-4-26B-A4B [Large language model]. Hugging Face. Available online at: https://huggingface.co/google/gemma-4-26B-A4B (Accessed May 15, 2026).

[B32] Gupta R. D. Morol M. K. Fahad N. Hosain M. T. Choya S. B. Z. Hossen M. J. (2025). “BRAINS: A Retrieval-Augmented System For Alzheimer's Detection And Monitoring” in 2025 international conference on machine learning and applications (ICMLA) (1473-1478). IEEE.

[B33] Gupta S. Singh P. Chang K. Qu L. Aggarwal M. Arun N. . (2021). Addressing catastrophic forgetting for medical domain expansion. arXiv [preprint]. arXiv:2103.13511.

[B34] Hartsock I. Rasool G. (2024). Vision-language models for medical report generation and visual question answering: a review. Front. Artif. Intell. 7:1430984. doi: 10.3389/frai.2024.1430984 PMC11611889 39628839

[B35] Hobson J. (2015). The Montreal cognitive assessment (MoCA). Occupat. Med. 65, 764–765. doi: 10.1093/occmed/kqv078 26644445

[B36] Hugging Face (2025). Huggingface.co. Available online at: https://huggingface.co/cross-encoder/ms-marco-MiniLM-L6-v2

[B37] Jack Jr C. R. Wiste H. J. Algeciras-Schimnich A. Figdore D. J. Schwarz C. G. Lowe V. J. . (2023). Predicting amyloid PET and tau PET stages with plasma biomarkers. Brain 146, 2029–2044. 10.1093/brain/awad042 PMC10151195 36789483

[B38] Jack C. R. Bennett D. A. Blennow K. Carrillo M. C. Dunn B. Haeberlein S. B. . (2018). NIA-AA research framework: toward a biological definition of Alzheimer's disease. Alzheimer's Dement. 14, 535–562. doi: 10.1016/j.jalz.2018.02.018 PMC5958625 29653606

[B39] Jack C. R. Bennett D. A. Blennow K. Carrillo M. C. Feldman H. H. Frisoni G. B. . (2016). A/T/N: an unbiased descriptive classification scheme for Alzheimer disease biomarkers. Neurology. 87, 539–547. doi: 10.1212/WNL.0000000000002923 PMC4970664 27371494

[B40] Jack C. R. Bernstein M. A. Fox N. C. Thompson P. Alexander G. Harvey D. . (2008). The Alzheimer's disease neuroimaging initiative (ADNI): MRI methods. J. Magn. Res. Imag. 27, 685–691. doi: 10.1002/jmri.21049 PMC2544629 18302232

[B41] Jahanshad N. Kochunov P. V. Sprooten E. Mandl R. C. Nichols T. E. Almasy L. . (2013). Multi-site genetic analysis of diffusion images and voxelwise heritability analysis: a pilot project of the ENIGMA-DTI working group. Neuroimage 81, 455–469. 10.1016/j.neuroimage.2013.04.061 PMC3729717 23629049

[B42] Jasodanand V. H. Bellitti M. Kolachalama V. B. (2025a). An AI-first framework for multimodal data in Alzheimer's disease and related dementias. Alzheimer's Demen. 21:e70719. doi: 10.1002/alz.70719 PMC12453944 40983947

[B43] Jasodanand V. H. Kowshik S. S. Puducheri S. Romano M. F. Xu L. Au R. . (2025b). AI-driven fusion of multimodal data for Alzheimer's disease biomarker assessment. Nat. Commun. 16:7407. doi: 10.1038/s41467-025-62590-4 PMC12339743 40789853

[B44] Jenkinson M. Beckmann C. F. Behrens T. E. J. Woolrich M. W. Smith S. M. (2012). FSL. Neuroimage 62, 782–790. doi: 10.1016/j.neuroimage.2011.09.015 21979382

[B45] Jeong D. P. Garg S. Lipton Z. C. Oberst M. (2024). “Medical adaptation of large language and vision-language models: Are we making progress?,” in Proceedings of the 2024 Conference on Empirical Methods in Natural Language Processing, 12143–12170.

[B46] Ji Z. Yu T. Xu Y. Lee N. Ishii E. Fung P. (2023). “Towards mitigating LLM hallucination via self reflection,” in Findings of the Association for Computational Linguistics: EMNLP 2023, pp. 1827–1843. doi: 10.18653/v1/2023.findings-emnlp.123

[B47] Jia S. Bit S. Jasodanand V. H. Liu Y. Kolachalama V. B. (2026). Agentic memory-augmented retrieval and evidence grounding for medical question-answering tasks. Int. J. Med. Inform. 212:106339. doi: 10.1016/j.ijmedinf.2026.106339 PMC13059660 41713127

[B48] Johnson J. Douze M. Jégou H. (2019). Billion-scale similarity search with GPUs. IEEE Transact. Big Data 7, 535–547. doi: 10.1109/TBDATA.2019.2921572

[B49] Johnson K. A. Sperling R. A. Gidicsin C. M. Carmasin J. S. Maye J. E. Coleman R. E. . (2013). Florbetapir (F18-AV-45) PET to assess amyloid burden in Alzheimer's disease dementia, mild cognitive impairment, and normal aging. Alzheimers Dement. 9(5S), S72–S83. 10.1016/j.jalz.2012.10.007 PMC3800236 23375563

[B50] Kueper J. K. Speechley M. Montero-Odasso M. (2018). The Alzheimer's disease assessment scale–cognitive subscale (ADAS-Cog): modifications and responsiveness in pre-dementia populations. A narrative review. J. Alzheimer's Dis. 63, 423–444. doi: 10.3233/JAD-170991 PMC5929311 29660938

[B51] Lam P. Zhu A. H. Gari I. B. Jahanshad N. Thompson P. M. (2020). 3D grid-attention networks for interpretable age and Alzheimer's disease prediction from structural MRI. arXiv [preprint] arXiv:2011.09115.

[B52] LaMontagne P. J. Benzinger T. L. Morris J. C. Keefe S. Hornbeck R. Xiong C. et al (2019). OASIS-3: longitudinal neuroimaging, clinical, and cognitive dataset for normal aging and Alzheimer disease. medrxiv, 2019–12.

[B53] Lau J. J. Gayen S. Ben Abacha A. Demner-Fushman D. (2018). A dataset of clinically generated visual questions and answers about radiology images. Scient. Data 5:180251. doi: 10.1038/sdata.2018.251 PMC6244189 30457565

[B54] Lee C. S. Lee A. Y. (2020). Clinical applications of continual learning machine learning. Lancet Digit. Health 2, e279–e281. doi: 10.1016/S2589-7500(20)30102-3 PMC8259323 33328120

[B55] Lewis P. Perez E. Piktus A. Petroni F. Karpukhin V. Goyal N. . (2020). Retrieval-augmented generation for knowledge-intensive NLP tasks. Adv. Neural Inf. Process. Syst. 33, 9459–9474.

[B56] Li C. Wong C. Zhang S. Usuyama N. Liu H. Yang J. . (2023). Llava-med: Training a large language-and-vision assistant for biomedicine in one day. Adv. Neural Inf. Process. Syst. 36, 28541–28564.

[B57] Lin C. Y. (2004). “Rouge: a package for automatic evaluation of summaries,” in Text Summarization Branches Out (pp. 74–81).

[B58] Lin P. J. Jiang Z. Liu Y. Li W. Qi Y. Sun J. . (2025). A vision-language foundation model for Alzheimer's disease diagnosis using MRI and clinical data. Alzheimer's Demen. 21:e71029. doi: 10.1002/alz.71029 PMC12745493 41457461

[B59] Liu H. Li C. Wu Q. Lee Y. J. (2023). Visual instruction tuning. Adv. Neural. Inf. Process Syst. 36, 34892–34916. doi: 10.52202/075280-1516

[B60] Lteif D. Jia S. Bit S. Kaliaev A. Mian A. Z. Small J. E. . (2026). Vision-language framework for multi-sequence brain magnetic resonance imaging. Medrxiv 2026-03. doi: 10.64898/2026.03.30.26349106

[B61] Marinescu R. V. Oxtoby N. P. Young A. L. Bron E. E. Toga A. W. Weiner M. W. . (2019). “TADPOLE challenge: accurate Alzheimer's disease prediction through crowdsourced forecasting of future data,” in International Workshop on Predictive Intelligence in Medicine (Cham: Springer International Publishing), pp. 1–10. doi: 10.1007/978-3-030-32281-6_1 PMC7315046 32587957

[B62] Mayeux R. Saunders A. M. Shea S. Mirra S. Evans D. Roses A. D. . (1998). Utility of the apolipoprotein E genotype in the diagnosis of Alzheimer's disease. New Engl. J. Med. 338, 506–511. doi: 10.1056/NEJM199802193380804 9468467

[B63] McDougall F. Edgar C. Mertes M. Delmar P. Fontoura P. Abi-Saab D. . (2021). Psychometric properties of the Clinical Dementia Rating-Sum of Boxes and other cognitive and functional outcomes in a prodromal Alzheimer's disease population. J. Prev. Alzheimers Dis. 8, 151–160. 10.14283/jpad.2020.73 33569561

[B64] Mehdipour Ghazi M. Nielsen M. Pai A. Cardoso M. J. Modat M. Ourselin S. . (2019). Training recurrent neural networks robust to incomplete data: application to Alzheimer's disease progression modeling. Med. Image Anal. 53, 39–46. doi: 10.1016/j.media.2019.01.004 30682584

[B65] Mistral AI (2023). mistralai/Mistral-7B-v0.1. Hugging Face. Available online at: https://huggingface.co/mistralai/Mistral-7B-v0.1

[B66] Nakamura A. Kaneko N. Villemagne V. L. Kato T. Doecke J. Doré V. . (2018). High performance plasma amyloid-β biomarkers for Alzheimer's disease. Nature 554, 249–254. 10.1038/nature25456 29420472

[B67] Nir T. M. Jahanshad N. Villalon-Reina J. E. Toga A. W. Jack C. R. Weiner M. W. . (2013). Effectiveness of regional DTI measures in distinguishing Alzheimer's disease, MCI, and normal aging. NeuroImage Clinical 3, 180–195. 10.1016/j.nicl.2013.07.006 PMC3792746 24179862

[B68] O'Bryant S. E. Waring S. C. Cullum C. M. Hall J. Lacritz L. Massman P. J. . (2008). Staging dementia using clinical dementia rating scale sum of boxes scores: a Texas Alzheimer's research consortium study. Arch. Neurol. 65, 1091–1095. doi: 10.1001/archneur.65.8.1091 PMC3409562 18695059

[B69] Oord A. V. D. Li Y. Vinyals O. (2018). Representation learning with contrastive predictive coding. arXiv preprint arXiv:1807.03748.

[B70] Papineni K. Roukos S. Ward T. Zhu W. J. (2002). “Bleu: a method for automatic evaluation of machine translation,” in Proceedings of the 40th Annual Meeting of the Association for Computational Linguistics (pp. 311–318). doi: 10.3115/1073083.1073135

[B71] Perkonigg M. Hofmanninger J. Herold C. J. Brink J. A. Pianykh O. Prosch H. . (2021). Dynamic memory to alleviate catastrophic forgetting in continual learning with medical imaging. Nat. Commun. 12:5678. doi: 10.1038/s41467-021-25858-z PMC8479083 34584080

[B72] Petrosian A. A. Prokhorov D. V. Lajara-Nanson W. Schiffer R. B. (2001). Recurrent neural network-based approach for early recognition of Alzheimer's disease in EEG. Clin. Neurophysiol. 112, 1378–1387. doi: 10.1016/S1388-2457(01)00579-X 11459677

[B73] Radford A. Kim J. W. Hallacy C. Ramesh A. Goh G. Agarwal S. . (2021). “Learning transferable visual models from natural language supervision,” in International conference on machine learning (PmLR), 8748–8763

[B74] Rahman S. Rahman M. M. Bhatt S. Sundararajan R. Faezipour M. (2025). NeuroNet-AD: a multimodal deep learning framework for multiclass Alzheimer's disease diagnosis. Bioengineering 12:1107. doi: 10.3390/bioengineering12101107 PMC12561078 41155106

[B75] Reimers N. Gurevych I. (2019). “Sentence-bert: sentence embeddings using siamese bert-networks,” in Proceedings of the 2019 Conference on Empirical Methods in Natural Language Processing and the 9th International Joint Conference on Natural Language Processing (EMNLP-IJCNLP) (pp. 3982–3992). doi: 10.18653/v1/D19-1410

[B76] Rieke N. Hancox J. Li W. Milletari F. Roth H. R. Albarqouni S. . (2020). The future of digital health with federated learning. NPJ Digital Med. 3:119. 10.1038/s41746-020-00323-1 PMC7490367 33015372

[B77] Rockwood K. Strang D. MacKnight C. Downer R. Morris J. C. (2000). Interrater reliability of the Clinical Dementia Rating in a multicenter trial. J. Am. Geriatr. Soc. 48, 558–559. 10.1111/j.1532-5415.2000.tb05004.x 10811551

[B78] Routier A. Marcoux A. Melo M. D. Guillon J. Samper-González J. Wen J. . (2019). “New advances in the Clinica software platform for clinical neuroimaging studies,” in OHBM 2019-Annual Meeting on Organization for Human Brain Mapping.

[B79] Seeher K. Cataldi R. Dua T. Kestel D. (2023). Inequitable access to dementia diagnosis and care in low-resource settings–a global perspective. Clin. Gerontol. 46, 133–137. doi: 10.1080/07317115.2022.2054391 35672953

[B80] Sellergren A. Gao C. Mahvar F. Kohlberger T. Jamil F. Traverse M. . (2026). Medgemma 1.5 technical report. arXiv [preprint]. arXiv:2604.05081

[B81] Singhal K. Tu T. Gottweis J. Sayres R. Wulczyn E. Amin M. . (2025). Toward expert-level medical question answering with large language models. Nat. Med. 31, 943–950. doi: 10.1038/s41591-024-03423-7 PMC11922739 39779926

[B82] Steiner A. Pinto A. S. Tschannen M. Keysers D. Wang X. Bitton Y. . (2024). Paligemma 2: A family of versatile vlms for transfer. arXiv [preprint]. arXiv:2412.03555.

[B83] Stripelis D. Gupta U. Saleem H. Dhinagar N. Ghai T. Anastasiou C. . (2024). A federated learning architecture for secure and private neuroimaging analysis. Patterns 5. 10.1016/j.patter.2024.101031 PMC11368680 39233693

[B84] Stripelis D. Saleem H. Ghai T. Dhinagar N. Gupta U. Anastasiou C. . (2021). “Secure neuroimaging analysis using federated learning with homomorphic encryption,” in 17th international symposium on medical information processing and analysis, Vol. 12088 (SPIE), 351–359.

[B85] Thirunavukarasu A. J. Ting D. S. J. Elangovan K. Gutierrez L. Tan T. F. Ting D. S. W. (2023). Large language models in medicine. Nat. Med. 29, 1930–1940. doi: 10.1038/s41591-023-02448-8 37460753

[B86] Thomopoulos S. I. Nir T. M. Villalon-Reina J. E. Zavaliangos-Petropulu A. Maiti P. Zheng H. . (2021). “Diffusion MRI metrics and their relation to dementia severity: effects of harmonization approaches,” in 17th international symposium on medical information processing and analysis, Vol. 12088 (SPIE), 166–179.

[B87] Tu T. Azizi S. Driess D. Schaekermann M. Amin M. Chang P. C. . (2024). Towards generalist biomedical AI. Nejm Ai 1:AIoa2300138.

[B88] Wang M. L. Zhang R. P. Wu W. J. Lu Y. Wei X. E. Sun Z. . (2026). Evaluation of large language models for diagnostic impression generation from brain MRI report findings: a multicenter benchmark and reader study. NPJ Digit. Med. 9:187. 10.1038/s41746-026-02380-4 PMC12929788 41571872

[B89] Wang T. Qiu R. G. Yu M. (2018). Predictive modeling of the progression of Alzheimer's disease with recurrent neural networks. Scientif. Rep. 8:9161. doi: 10.1038/s41598-018-27337-w PMC6003986 29907747

[B90] Wood D. A. Guilhem E. Kafiabadi S. Busaidi A. A. Dissanayake K. Hammam A. . (2024). A self-supervised text-vision framework for automated brain abnormality detection. arXiv [preprint] *arXiv:2405.02782*.

[B91] Woolrich M. W. Jbabdi S. Patenaude B. Chappell M. Makni S. Behrens T. . (2009). Bayesian analysis of neuroimaging data in FSL. Neuroimage 45, S173–S186. 10.1016/j.neuroimage.2008.10.055 19059349

[B92] World Health Organization (2020). Dementia Fact Sheet. Available online at: https://www.who.int/news-room/fact-sheets/detail/dementia (Accessed May 15, 2026).

[B93] Wu C. Lei J. Zheng Q. Zhao W. Lin W. Zhang X. . (2023). Can gpt-4v (ision) serve medical applications? Case studies on gpt-4v for multimodal medical diagnosis. arXiv [preprint]. arXiv:2310.09909.

[B94] Wu C. Zhang X. Zhang Y. Hui H. Wang Y. Xie W. (2025). Towards generalist foundation model for radiology by leveraging web-scale 2D and 3D medical data. Nat. Commun. 16:7866. doi: 10.1038/s41467-025-62385-7 PMC12375113 40849424

[B95] Yang Z. Li L. Lin K. Wang J. Lin C. C. Liu Z. . (2023). The dawn of LMMS: preliminary explorations with gpt-4v (ision). arXiv [preprint]. arXiv:2309.17421.

[B96] Yi H. Qin Z. Lao Q. Xu W. Jiang Z. Wang D. . (2023). Towards general purpose medical ai: Continual learning medical foundation model. arXiv [preprint]. arXiv:2303.06580.

[B97] Zavaliangos-Petropulu A. Nir T. M. Thomopoulos S. I. Reid R. I. Bernstein M. A. Borowski B. . (2019). Diffusion MRI indices and their relation to cognitive impairment in brain aging: the updated multi-protocol approach in ADNI3. Front. Neuroinform. 13:2. doi: 10.3389/fninf.2019.00002 PMC6390411 30837858

[B98] Zhang J. Huang J. Jin S. Lu S. (2024). Vision-language models for vision tasks: a survey. IEEE Trans. Pattern Anal. Mach. Intell. 46, 5625–5644. doi: 10.1109/TPAMI.2024.3369699 38408000

[B99] Zhang K. Zhou R. Adhikarla E. Yan Z. Liu Y. Yu J. . (2024). A generalist vision–language foundation model for diverse biomedical tasks. Nat. Med. 30, 3129–3141. doi: 10.1038/s41591-024-03185-2 PMC12581140 39112796

[B100] Zhang S. Chattopadhyay T. Thomopoulos S. I. Ambite J. L. Thompson P. M. Ver Steeg G. (2025). “Diffusion bridge models for 3d medical image translation,” in 2025 47th annual international conference of the IEEE engineering in medicine and biology society (EMBC) (IEEE), 1–7. 10.1109/EMBC58623.2025.11252839 41336848

[B101] Zhang T. Kishore V. Wu F. Weinberger K. Q. Artzi Y. (2019). BERTScore: evaluating text generation with bert. arXiv preprint arXiv:1904.09675.

[B102] Zhao W.X. Zhou K. Li J. Tang T. Wang X. Hou Y. . (2023). A survey of large language models. Front. Comput. Sci. 20:2012627.

