# Supplementary material for: MEMOIR-VLM—a multimodal vision-language model for Alzheimer's disease classification and question answering
Source: Front Comput Neurosci. 2026 Oct 1;20:1902258. doi: 10.3389/fncom.2026.1902258 (PMC13668829; doi:10.3389/fncom.2026.1902258)
Supplement: Supplementary file 1 [file Data_Sheet_1.pdf]

1. Prompt Engineering

1.1 Diagnosis Prompt Template

The exact prompt sent to Mistral 7B for diagnosis prediction (wrapped in [INST]...[/INST]):

DX_PROMPT

```
[INST] You are a clinical neuroimaging AI. Based on the
similar subjects retrieved from the ADNI database, predict
the diagnosis for the query subject.

Context from similar subjects:
{context}

Answer in this exact format (first line MUST be the diagnosis):
Diagnosis: <CN or MCI or Dementia>
Reason: <one sentence explanation> [/INST]
```

1.2 Captioning Prompt Template

The exact prompt sent to Mistral 7B for captioning (wrapped in [INST]...[/INST]):

CAPTIONING_PROMPT

```
[INST] You are a clinical neuroimaging AI assistant analyzing
brain MRI and DTI scans for Alzheimer's Disease research.

Given a brain scan, a retrieval system found the following
similar subjects from the training database:

  {context}

Based on these similar cases, generate a concise clinical
description for the query subject. Include:
- Likely diagnosis (CN, MCI, or Dementia) and confidence
- Key clinical characteristics inferred from similar subjects
- Notable patterns (e.g., age group, amyloid status trends) [/INST]
```

Note that {context} is replaced at runtime with the captions of the top-k retrieved training subjects (after cross-encoder reranking). The LLM never sees the actual brain images and only sees the text captions of similar subjects found via embedding-space retrieval.

2. Example from the Pipeline

The following examples show the complete input to output flow for each VQA application, including the exact prompt sent to Mistral 7B and the model's response.

2.1 Example 1: Diagnosis VQA — CN Subject

Query subject: 82-year-old male, True DX: CN

Step 0 — Model inputs: The following data is fed into the frozen multi-modal encoders to produce the 512-d fused embedding z_f . This subject has T1w MRI only (no DTI-FA).

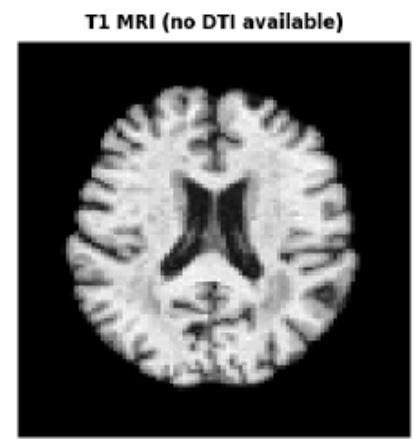

Fig: Axial T1w MRI slice (mid-brain) for CN subject. This slice from a 91×109×91 volume is the input to the 3D ResNet-18 T1w encoder. No DTI-FA scan is available for this subject so the DTI-FA modality slot is masked out in the attention fusion.

Clinical features input to the clinical encoder for the subject. These 7 values are each passed through a dedicated MLP sub-encoder, concatenated, and projected to 512-d.

Feature	Value	Feature	Value
Age	81.6	MMSE	29.0
CDR-SB	0.5	MOCA	— (default 25.0)
ADAS-11	2.67	APOE	e3/e3
ADAS-13	4.67		

The T1 volume goes through the 3D ResNet-18 → $z_{T1} \in R^{512}$. The clinical features go through the Clinical MLP → $z_{clin} \in R^{512}$. Since DTI is missing, the attention fusion attends only over $[z_{T1}, z_{clin}]$ to produce $z_f \in R^{512}$.

Step 1 — FAISS Retrieval: The query subject's 512-d fused embedding retrieves 50 candidates from the training index. Cross-encoder reranking selects the top-5:

Rank	DX	Age	FAISS sim	Rerank score
1	CN	82	0.995	9.12
2	CN	81	0.997	8.67
3	CN	84	0.996	8.64
4	CN	77	0.993	8.44
5	CN	77	0.994	8.33

Step 2 — Construct Prompt: The retrieved subjects' captions are concatenated and inserted into the DX_PROMPT template. The full prompt sent to Mistral 7B:

Full Prompt Sent to Mistral 7B

```
[INST] You are a clinical neuroimaging AI. Based on the
similar subjects retrieved from the ADNI database, predict
the diagnosis for the query subject.

Context from similar subjects:
Subject 1: 82-year-old male subject diagnosed with Cognitively Normal (CN).
  Clinical scores: MMSE=30.0, CDR-SB=0.0, ADAS-11=3.0, ADAS-13=6.0.
  APOE genotype: e3/e3.
  Imaging: T1 MRI and DTI FA available.
Subject 2: 81-year-old male subject diagnosed with Cognitively Normal (CN).
  Clinical scores: MMSE=29.0, CDR-SB=0.0, ADAS-11=7.0, ADAS-13=11.0.
  APOE genotype: e3/e3.
  Imaging: T1 MRI available.
Subject 3: 84-year-old male subject diagnosed with Cognitively Normal (CN).
  Clinical scores: MMSE=28.0, CDR-SB=0.5. APOE genotype: e3/e3.
  Imaging: T1 MRI available.
Subject 4: 77-year-old male subject diagnosed with Cognitively Normal (CN).
  Clinical scores: MMSE=28.0, CDR-SB=0.0, ADAS-11=9.3, ADAS-13=13.3.
  APOE genotype: e3/e3.
  Imaging: T1 MRI and DTI FA available.
Subject 5: 77-year-old male subject diagnosed with Cognitively Normal (CN).
  Clinical scores: MMSE=30.0, CDR-SB=0.0, MoCA=22.0.
  APOE genotype: e3/e3.
  Imaging: T1 MRI and DTI FA available.

Answer in this exact format (first line MUST be the diagnosis):
Diagnosis: <CN or MCI or Dementia>
Reason: <one sentence explanation> [/INST]
```

Step 3 — LLM Response:

"Diagnosis: CN

Reason: All five similar subjects are diagnosed as Cognitively Normal (CN) with high MMSE scores (28–30) and minimal CDR-SB scores (0.0–0.5), consistent with preserved cognitive function."

Step 4 — Parse: Regex matches Diagnosis: CN → predicted CN. **Correct.**

2.2 Example 2: Diagnosis VQA — MCI Subject

Query subject: 85-year-old male, True DX: MCI
Step 0 — Model inputs: This subject has both T1w MRI and DTI FA scans, so all three encoder branches are active.

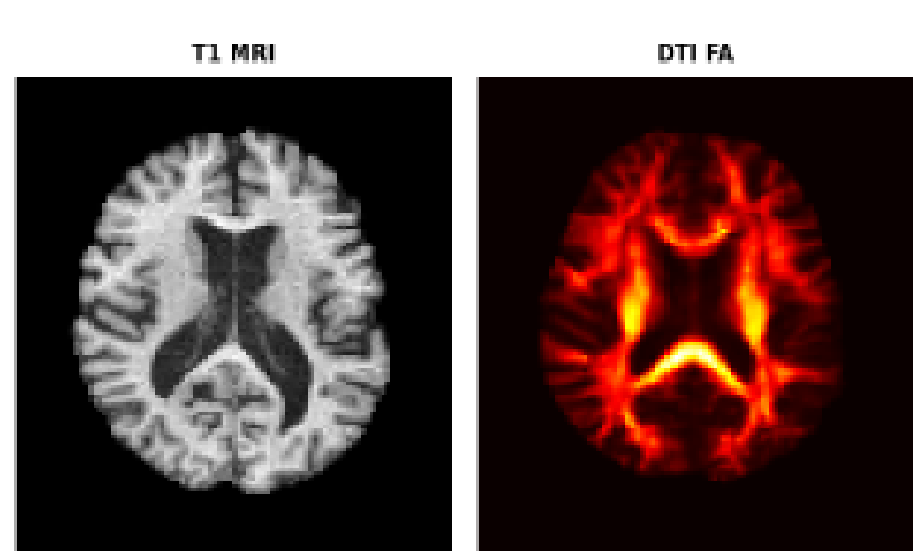

Fig: Axial T1w MRI slice and DTI-FA slice (mid-brain) for MCI subject. **Left:** T1w structural scan input to the T1w ResNet-18. **Right:** DTI-FA map input to the DTI-FA ResNet-18. Both are 91×109×91 volumes.

Clinical features input to the clinical encoder for the subject. These 7 values are each passed through a dedicated MLP sub-encoder, concatenated, and projected to 512-d.

Feature	Value	Feature	Value
Age	84.8	MMSE	27.0
CDR-SB	1.0	MOCA	24.0
ADAS-11	12.0	APOE	e3/e3
ADAS-13	18.0		

Here all three modalities contribute: z_{T1}, z_{DTI} , and z_{clin} are fused via attention to produce z_f. Note the clinical scores already show early cognitive decline: CDR-SB=1.0, ADAS-13=18.0(elevated), MoCA=24.0 (borderline).

Step 1 — FAISS Retrieval: All 5 retrieved subjects are MCI, ages 85–85.

Step 2 — Construct Prompt: The retrieved subjects' captions are concatenated and inserted into the DX_PROMPT template. The full prompt sent to Mistral 7B:

Full Prompt Sent to Mistral 7B

```
Subject 1: 85-year-old male subject diagnosed with Mild Cognitive Impairment (MCI).
  Clinical scores: MMSE=28.0, CDR-SB=1.5, ADAS-11=10.3, ADAS-13=18.3.
  APOE genotype: e3/e3. Imaging: T1 MRI and DTI FA available.
Subject 2: 85-year-old male subject diagnosed with Mild Cognitive Impairment (MCI).
  Clinical scores: MMSE=30.0, CDR-SB=1.0, ADAS-11=9.0, ADAS-13=14.0.
  APOE genotype: e3/e3. Imaging: T1 MRI available.
Subject 3: 84-year-old male subject diagnosed with Mild Cognitive Impairment (MCI).
  Clinical scores: MMSE=29.0, CDR-SB=1.5, ADAS-11=11.7, ADAS-13=18.0, MoCA=23.0.
  APOE genotype: e3/e3. Imaging: T1 MRI and DTI FA available.
Subject 4: 85-year-old male subject diagnosed with Mild Cognitive Impairment (MCI).
  Clinical scores: MMSE=28.0, CDR-SB=1.0, ADAS-11=8.0, ADAS-13=12.0.
  APOE genotype: e3/e3. Imaging: T1 MRI available.
Subject 5: 85-year-old male subject diagnosed with Mild Cognitive Impairment (MCI).
  Clinical scores: MMSE=29.0, CDR-SB=1.0, ADAS-11=9.3, ADAS-13=16.7.
  APOE genotype: e3/e4. Imaging: T1 MRI and DTI FA available.
```

Step 3 — LLM Response:

"Diagnosis: MCI

Reason: All five similar subjects are diagnosed with MCI, with MMSE scores 28–30, CDR-SB 1.0–1.5, and elevated ADAS-13 scores (12–18) indicating early cognitive decline."

Step 4 — Parse: Regex matches Diagnosis: MCI → predicted MCI. **Correct.**

2.3 Example 3: Diagnosis VQA — Dementia Subject

Query subject: 81-year-old male, True DX: Dementia
Step 0 — Model inputs: This subject has both T1w MRI and DTI FA scans, so all three encoder branches are active.

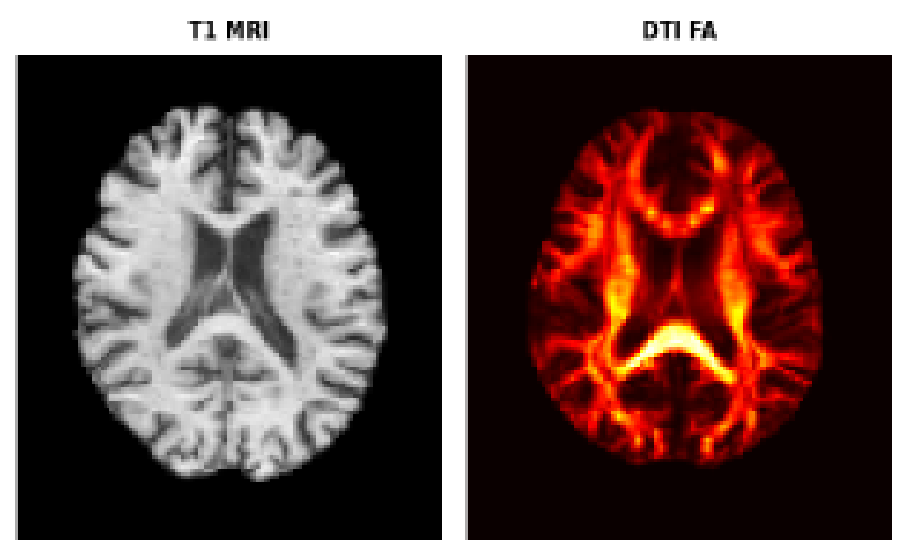

Fig: Axial T1w MRI slice and DTI-FA slice (mid-brain) for MCI subject. **Left:** T1w structural scan input to the T1w ResNet-18. **Right:** DTI-FA map input to the DTI-FA ResNet-18. Both are 91×109×91 volumes.

Clinical features input to the Clinical Encoder for the subject. These 7 values are each passed through a dedicated MLP sub-encoder, concatenated, and projected to 512-d.

Feature	Value	Feature	Value
Age	81.0	MMSE	16.0
CDR-SB	5.0	MOCA	— (default 25.0)
ADAS-11	33.67	APOE	e3/e4
ADAS-13	46.67		

Here all three modalities contribute: z_{T1}, z_{DTI} , and z_{clin} are fused via attention to produce z_f. The clinical profile is strikingly different from the CN and MCI examples: MMSE dropped from 29→27→16, ADAS-13 from 4.67→18→46.67, and CDR-SB from 0.5→1.0→5.0 across the three severity levels.

Step 1 — FAISS Retrieval: All 5 retrieved subjects are Dementia, ages 79–84.

Step 2 — Construct Prompt: The retrieved subjects' captions are concatenated and inserted into the DX_PROMPT template. The full prompt sent to Mistral 7B:

Full Prompt Sent to Mistral 7B

```
Subject 1: 82-year-old male subject diagnosed with Dementia.
  Clinical scores: MMSE=21.0, CDR-SB=5.0, ADAS-11=24.0, ADAS-13=34.0.
  APOE genotype: e3/e4.
  Imaging: T1 MRI and DTI FA available.
Subject 2: 82-year-old male subject diagnosed with Dementia.
  Clinical scores: MMSE=19.0, CDR-SB=4.5, ADAS-11=29.0, ADAS-13=42.7, MoCA=19.0.
  APOE genotype: e3/e4.
  Imaging: T1 MRI and DTI FA available.
Subject 3: 84-year-old male subject diagnosed with Dementia.
  Clinical scores: MMSE=24.0, CDR-SB=4.5, ADAS-11=20.0, ADAS-13=32.0.
  APOE genotype: e3/e4.
  Imaging: T1 MRI available.
Subject 4: 83-year-old male subject diagnosed with Dementia.
  Clinical scores: MMSE=22.0, CDR-SB=5.5, ADAS-11=24.7, ADAS-13=38.0.
  APOE genotype: e3/e4.
  Imaging: T1 MRI available.
Subject 5: 79-year-old male subject diagnosed with Dementia.
  Clinical scores: MMSE=26.0, CDR-SB=4.0, ADAS-11=15.3, ADAS-13=24.0, MoCA=22.0.
  APOE genotype: e3/e4.
  Imaging: T1 MRI and DTI FA available.
```

Step 3 — LLM Response:

"Diagnosis: Dementia

Reason: All five similar subjects are diagnosed with Dementia, with low MMSE scores (19–26), elevated CDR-SB (4.0–5.5), and high ADAS-13 scores (24–43), all carrying the e3/e4 APOE risk genotype."

Step 4 — Parse: Regex matches Diagnosis: Dementia → predicted Dementia. **Correct.**

2.4 Example 4: Captioning

Query subject: 81-year-old male, True DX: Dementia
The same 5 retrieved subjects from **2.3 Example 3** are used as context, but with the CAPTIONING_ PROMPT template instead. The LLM generates a free-form clinical description:

Generated Caption:

"Based on the provided clinical scores and similar subjects, it is likely that the query subject has Dementia. The confidence level is high, given that all five similar subjects were also diagnosed with Dementia.
Key clinical characteristics inferred from similar subjects include:
• Age group: The query subject and similar subjects are all elderly males, with ages ranging from 79 to 84 years old.
• Cognitive function: MMSE scores 19–26 indicating moderate cognitive impairment. MoCA 19–22 confirm deficits.
• Disease severity: CDR-SB 4.0–5.5 and ADAS-13 24–43 indicate significant functional and memory impairment.
• Notable patterns: All similar subjects carry the APOE e3/e4 genotype, a known genetic risk factor for Alzheimer's disease."

2.5 Example 5: Open Ended VQA

Query subject: 82-year-old male, True DX: CN

Open-ended questions use the VQA_PROMPT template, which includes a {question} field:

Question 1

"What clinical characteristics suggest this diagnosis?"

Mistral 7B Output

"Based on the provided context, the clinical characteristics that suggest a diagnosis of CN include: (1) Age range: 77–84 years old. (2) High MMSE scores: 28–30, indicating intact global cognition. (3) Low CDR-SB scores: 0.0–0.5, no significant functional impairment. (4) APOE genotype: all subjects have e3/e3, the most common and carries no elevated Alzheimer's risk."

A key factor to note here is that the LLM never sees the actual brain images. Instead, the brain scans are encoded into 512-d embeddings by the frozen encoders, FAISS retrieves similar training subjects in embedding space, and only the text captions of those retrieved subjects are passed to Mistral 7B as context. The LLM reasons over clinical metadata (ages, scores, genotypes) of similar cases and not over raw pixel/voxel data.
